# A multiplex oligonucleotide ligation-PCR as a complementary tool for subtyping of *Salmonella* Typhimurium

**DOI:** 10.1007/s00253-015-6831-7

**Published:** 2015-07-25

**Authors:** Véronique Wuyts, Wesley Mattheus, Nancy H. C. Roosens, Kathleen Marchal, Sophie Bertrand, Sigrid C. J. De Keersmaecker

**Affiliations:** Department of Microbial and Molecular Systems, KU Leuven, Kasteelpark Arenberg 20 bus 2460, 3001 Leuven, Belgium; Department of Plant Biotechnology and Bioinformatics, Ghent University, Technologiepark 927, 9052 Ghent, Belgium; Platform Biotechnology and Molecular Biology, Scientific Institute of Public Health (WIV-ISP), Juliette Wytmanstraat 14, 1050 Brussels, Belgium; National Reference Centre for Salmonella and Shigella, Bacterial Diseases Division, Communicable and Infectious Diseases, Scientific Institute of Public Health (WIV-ISP), Juliette Wytmanstraat 14, 1050 Brussels, Belgium; Department of Information Technology, Ghent University, IMinds, Gaston Crommenlaan 8, 9050 Ghent, Belgium

**Keywords:** MOL-PCR, *Salmonella* Typhimurium, Subtyping, Luminex, Microsphere suspension array

## Abstract

Subtyping below the serovar level is essential for surveillance and outbreak detection and investigation of *Salmonella enterica* subsp. *enterica* serovar Typhimurium (*S.* Typhimurium) and its monophasic variant 1,4,[5],12:i:- (*S*. 1,4,[5],12:i:-), frequent causes of foodborne infections. In an attempt to overcome the intrinsic shortcomings of currently used subtyping techniques, a multiplex oligonucleotide ligation-PCR (MOL-PCR) assay was developed which combines different types of molecular markers in a high-throughput microsphere suspension array. The 52 molecular markers include prophage genes, amplified fragment length polymorphism (AFLP) elements, *Salmonella* genomic island 1 (SGI1), allantoinase gene *allB*, MLVA locus STTR10, antibiotic resistance genes, single nucleotide polymorphisms (SNPs) and phase 2 flagellar gene *fljB*. The in vitro stability of these markers was confirmed in a serial passage experiment. The validation of the MOL-PCR assay for subtyping of *S*. Typhimurium and *S*. 1,4,[5],12:i:- on 519 isolates shows that the method is rapid, reproducible, flexible, accessible, easy to use and relatively inexpensive. Additionally, a 100 % typeability and a discriminatory power equivalent to that of phage typing were observed, and epidemiological concordance was assessed on isolates of 2 different outbreaks. Furthermore, a data analysis method is provided so that the MOL-PCR assay allows for objective, computerised data analysis and data interpretation of which the results can be easily exchanged between different laboratories in an international surveillance network.

**Electronic supplementary material:**

The online version of this article (doi:10.1007/s00253-015-6831-7) contains supplementary material, which is available to authorized users.

## Introduction

*Salmonella*, one of the major causes of foodborne infections worldwide, is reported to be responsible for about 85,000 human illnesses each year in Europe, with an approximate hospitalisation rate of 36 %, and every year, nontyphoidal salmonellosis is accountable for 59 deaths (European Food Safety Authority (EFSA), European Centre for Disease Prevention and Control (ECDC) (2015)).

*Salmonella* is a complex genus with 2 species, 6 subspecies and 2659 serovars (Grimont and Weill 2007; Issenhuth-Jeanjean et al. 2014). In Europe, near 29 % of the reported human salmonellosis cases are attributed to *Salmonella enterica* subsp. *enterica* serovar Typhimurium (*S.* Typhimurium) and its monophasic variant *S. enterica* subsp. *enterica* serovar 1,4,[5],12:i:- (*S*. 1,4,[5],12:i:-), making them the second and third most commonly reported serovars after *S. enterica* subsp. *enterica* serovar Enteritidis (about 40 % of the reported cases) (European Food Safety Authority (EFSA), European Centre for Disease Prevention and Control (ECDC) (2015)). Once serotyped as *S*. Typhimurium or *S*. 1,4,[5],12:i:-, different techniques are applied for further subtyping of the isolate below the serovar level, which is necessary for surveillance, outbreak detection or outbreak investigation. The classical phage typing technique is nowadays complemented with molecular methods for which pulsed-field gel electrophoresis (PFGE) is considered the gold standard. Other molecular methods used for subtyping of *S*. Typhimurium are multiple-locus variable-number of tandem repeats analysis (MLVA), multilocus sequence typing (MLST) and clustered regularly interspaced short palindromic repeats (CRISPR) genotyping. Advantages and disadvantages of each of these techniques have been discussed previously (Boxrud 2010; Sabat et al. 2013; Wattiau et al. 2011) and, although proven to have additional value for subtyping, each of these techniques has one or more attributes that do not correspond to the ideal subtyping method, which should be inexpensive, rapid, straightforward to execute, highly discriminative, robust, universally applicable for a wide range of bacterial pathogens and generating objective data, which can be easily interpreted and transferred between different laboratories (Sabat et al. 2013; Wattiau et al. 2011).

In recent years, whole genome sequencing (WGS) has been introduced and promoted as the ultimate subtyping method for each pathogen (Sabat et al. 2013), and several *Salmonella* epidemiological investigations reporting its added value have been published (some recent examples: Angelo et al. 2015; Ashton et al. 2015; Deng et al. 2015; Octavia et al. 2015). However, although sequencing may eventually have similar costs as other subtyping methods, the turn-around-time from sample to completely analysed data for this technology is not to be neglected (>24 hours). Moreover, there are still many laboratories which do not have the resources for the substantial investments, both at the level of equipment as of data analysis, nor have the required high-throughput that is needed to obtain these low sequencing costs (European Food Safety Authority (EFSA) (2014)). Therefore, new molecular assays which complement existing subtyping methods and which do not demand high-end equipment nor complex data analysis still have a role to play in these laboratories before WGS will become the gold standard in all European National Reference Laboratories and Centres.

Molecular subtyping of *S*. Typhimurium below the serovar level requires multiple markers and for the time-effectiveness of the assay, these markers should be combined in a multiplex assay. A multiplex prophage marker subtyping method was developed by Fang et al. (2012). In this assay, 30 prophage-related markers were amplified in 2 separate 15-plex PCRs and the amplicons were analysed with the Luminex xMAP technology in a direct hybridisation assay. In this type of assay, the fluorescent amplicons from a multiplex PCR are hybridised to marker-specific probes, which are covalently coupled to carboxylated microspheres. As different probes are linked to differently coloured microspheres, absence or presence of a marker can be detected in a Luminex device by determining the microsphere colour, and thus the marker, and checking the presence of a hybridised amplicon through its fluorescence. Further refinement of the assay of Fang et al. (2012) would necessitate inclusion of more markers, which could be challenging due to the limited multiplexing capacity of a PCR. Multiplex ligation-dependent probe amplification (MLPA) (Schouten et al. 2002) allows a higher multiplexing capacity than multiplex PCR, since the multiplex phase is a ligation which is then followed by a singleplex PCR. However, MLPA requires an overnight hybridisation step, which makes it a relatively extended protocol. For the rapid detection of biothreat agents, Deshpande et al. (2010) shortened the MLPA protocol by performing the hybridisation and ligation in a thermal cycling step and introduced the Luminex xTAG technology, based on microspheres with anti-TAG sequences pre-coupled to their surface, for the analysis of the PCR products, where the original MLPA protocol relies on fragment sizing by electrophoresis. The resulting multiplex oligonucleotide ligation-PCR (MOL-PCR) allows the detection of a combination of different types of molecular markers, such as single nucleotide polymorphisms (SNPs), unique sequences, insertions and deletions.

MOL-PCR and MLPA have already been described for characterisation and subtyping of pathogens (Bergval et al. 2012; Beyene et al. 2009; Cornelius et al. 2014; Pham Thanh et al. 2013; Stucki et al. 2012; Thierry et al. 2013), but also for the detection of bacteria (Berning et al. 2014; Chung et al. 2012; Deshpande et al. 2010) and viruses (De Smet et al. 2012; Reijans et al. 2008; Theelen et al. 2010) and for diagnosis of human genetic diseases (Garin et al. 2014; Kasatkar et al. 2014; Marcinkowska-Swojak et al. 2014; Schouten et al. 2002; Slater et al. 2004; Xu et al. 2013). Here, we describe a MOL-PCR for subtyping of *S.* Typhimurium that attempts to overcome the major disadvantages of the currently used subtyping methods, including those of previously described Luminex assays for *Salmonella*. The assay combines markers including prophage genes, amplified fragment length polymorphism (AFLP) elements, *Salmonella* genomic island 1 (SGI1), allantoinase gene *allB*, MLVA locus STTR10, antibiotic resistance, SNPs and phase 2 flagellar gene *fljB*. We elaborate on the development of the assay, report the validation of the subtyping method on a large collection of *S*. Typhimurium and *S*. 1,4,[5],12:i:- isolates and provide an analysis method for use in routine subtyping.

## Materials and methods

### Bacterial isolates

All *S*. Typhimurium and *S*. 1,4,[5],12:i:- isolates were received from the Belgian National Reference Centre for *Salmonella* and *Shigella* and are listed in Data set S1. All isolates are available upon request. The validation panel of 519 human *S*. Typhimurium and *S*. 1,4,[5],12:i:- isolates (S0001-S0519 in Data set S1) collected in Belgium in the period 2010–2013 contained 33 different phage types, including 29 non-typable (NT) and 39 reacts-but-does-not-conform (RDNC) isolates, and covered 168 distinct MLVA profiles. Additionally, 13 *S*. Typhimurium and *S*. 1,4,[5],12:i:- isolates related to 2 different Belgian outbreaks were used in this study. Out-group isolates were isolated around the same time as the corresponding outbreak. Phage typing (Threlfall and Frost 1990) and MLVA (Larsson et al. 2009; Lindstedt et al. 2004) were performed by the Belgian National Reference Centre for *Salmonella* and *Shigella*.

### Pulsed-field gel electrophoresis

PFGE (Hunter et al. 2005; Ribot et al. 2006) was performed according to the PulseNet Europe protocol (http://www.pulsenetinternational.org/networks/europe). Genomic DNA was digested with *Xba*I restriction enzyme and *Xba*I-digested genomic DNA of *S. enterica* subsp. *enterica* serovar Braenderup was used as a size marker. For the PFGE analysis, 53 *S*. Typhimurium and *S*. 1,4,[5],12:i:- isolates representing one of the 3 most frequently observed MOL-PCR profiles were selected from the validation panel. This selection was made in order to include a high variability of phage types in combination with MLVA profiles. The 53 isolates were run on 4 separate gels. PFGE patterns were analysed with Bionumerics (version 7.1; Applied Maths). A dendrogram was created with following similarity-based clustering parameters: unweighted pair group method using arithmetic averages (UPGMA) with Dice similarity coefficient and 1.0 % optimisation and tolerance settings.

### DNA isolation

DNA template was prepared by heat lysis. Hereto, a single colony from an overnight (14 to 20 h) culture at 37 °C on LB agar (Merck Millipore) was dissolved in 50 μl sterile de-ionised water and incubated at 100 °C in a heating block for 10 min. After cooling for a minimum of 5 min at 4 °C and centrifugation for 10 min at 11,000×*g*, the supernatant was stored at −20 °C and used for further analysis.

### Selection of molecular markers

The first step in the selection of molecular markers consisted of a literature study (Boyd et al. 2001; Drahovská et al. 2007; Fang et al. 2012; Hu et al. 2002, 2006; Lan et al. 2007; Lindstedt et al. 2004; Mikasová et al. 2005; Pang et al. 2012; Ross and Heuzenroeder 2005; Rychlík et al. 2008) to identify molecular markers which could potentially discriminate between *S*. Typhimurium isolates and to find molecular markers which could serve as internal positive control of the *Salmonella* DNA template. In the second step, the molecular markers that could be informative through presence or absence, i.e., SAL-1 up to SAL-55 in Table S1, were screened by PCR and gel electrophoresis. For this PCR screening, 27 *S*. Typhimurium and *S*. 1,4,[5],12:i: isolates of common phage types in Belgium (DT12, DT104, DT120, DT193, DT195 and U302) (Bertrand et al. 2014) were selected and complemented with 1 NT isolate, 1 RDNC isolate and 1 isolate of an uncommon phage type in Belgium (DT35) (isolates S0001-S0030 in Data set S1). DNA was isolated by heat lysis as described above, except that 300 μl sterile de-ionised water was used instead of 50 μl. The PCR was performed in a final reaction volume of 25 μl including 1× DreamTaq buffer (Thermo Fisher Scientific), 200 to 500 nM of forward and reverse primer (Table S1), 200 μM of each dNTP (Thermo Fisher Scientific), 0.625 U DreamTaq DNA polymerase (Thermo Fisher Scientific) and 2 μl DNA template. The following protocol was run in a thermal cycler: 10 min at 95 °C, 35 cycles of 30 s at 94 °C, 30 s at 45 to 60 °C (Table S1) and 1 min at 72 °C, 10 min at 72 °C. PCR products were visualised by agarose gel electrophoresis with ethidium bromide staining.

### Probe design

Upstream and downstream probes were designed with Visual OMP (version 7.6.58.0; DNA Software) as previously described (Wuyts et al. 2015). For markers for which a primer pair was reported in literature, it was attempted to take the forward or reverse primer as the target-specific sequence of the upstream probe. For SNP markers, except for an internal positive control marker, a probe with the wild-type allele was also included in the assay.

### MOL-PCR assay protocol

The MOL-PCR assay parameters were optimised as previously described (Wuyts et al. 2015).

The selected markers were divided over three MOL-PCRs, i.e., MOL-PCR_1, MOL-PCR_2 and MOL-PCR_SNP, as listed in Table 1.

**Table 1:** Type of markers and division over MOL-PCRs in the subtyping assay

MOL-PCR	Internal PC	Prophage	AFLP	SGI1	Antibiotic resistance	SNP	Other	Plex
MOL-PCR_1	*invA*	8	2	2	4	-	STTR10	20
	*rpoB*						*allB*	
MOL-PCR_2	*rpoB*	9	10	-	1	-	*fljB*	22
MOL-PCR_SNP	-	-	-			11	-	11^a^
Total	2	17	12	2	5	11	3	50 + 2

*AFLP* amplified fragment length polymorphism, *MOL-PCR* multiplex oligonucleotide ligation-PCR, *PC* positive control, *SGI1 Salmonella* genomic island 1, *SNP* single nucleotide polymorphism

^a^MOL-PCR_SNP includes 11 SNP alleles and 11 corresponding wild-type alleles and as such, 22 different regions of MagPlex-TAG microspheres are included in MOL-PCR_SNP

The multiplex oligonucleotide ligation reaction occurred in a 10 μl volume with 1× *Taq* DNA ligase reaction buffer (New England BioLabs), 2 nM of each probe (Tables S2, S3 and S4, Eurogentec), 2 U of *Taq* DNA ligase (New England BioLabs), 2 μl of DNA template and nuclease-free distilled water (Thermo Fisher Scientific). The thermal cycling programme (Swift MaxPro, Esco) included 10 min of denaturation at 95 °C followed by 30 cycles of 25 s at 94 °C and 30 s at 58 °C.

The singleplex PCR was performed in a final volume of 10 μl composed of 1× HotStarTaq PCR buffer (Qiagen), 125 nM T7 primer (TAATACGACTCACTATAGGG, Eurogentec), 500 nM 5′-biotin-T3 primer (ATTAACCCTCACTAAAGGGA, Eurogentec), 200 μM of each dNTP (Thermo Fisher Scientific), 0.25 U HotStarTaq DNA polymerase (Qiagen) and 3 μl of ligase product. The PCR protocol was 15 min at 95 °C, 35 cycles of 30 s at 94 °C, 30 s at 60 °C and 30 s at 72 °C, 5 min at 72 °C (Swift MaxPro, Esco).

The necessary regions (Tables S2, S1 and S4) of MagPlex-TAG microspheres (Luminex) were diluted to 750 microspheres of each region per reaction in 1.25× Tm hybridisation buffer (0.125 M Tris-HCl pH 8.0 (Sigma), 0.25 M NaCl (Sigma), 0.1 % Triton X-100 (Sigma) in nuclease-free distilled water (Thermo Fisher Scientific), sterilised by filtration (0.2 μm)). In a total volume of 25 μl, 5 μl of the PCR product was combined with the microsphere mix to a final concentration of 1× Tm hybridisation buffer (0.1 M Tris-HCl pH 8.0 (Sigma), 0.2 M NaCl (Sigma), 0.08 % Triton X-100 (Sigma) in nuclease-free distilled water (Thermo Fisher Scientific), sterilised by filtration (0.2 μm)). In a thermal cycler, the samples were denatured for 90 s at 96 °C and hybridisation to anti-TAGs on the microspheres occurred for 30 min at 37 °C. Hundred microlitres of a reporter mix including 4 μg/ml of streptavidin-R-phycoerythrin (SAPE) (Thermo Fisher Scientific) in 1× Tm hybridisation buffer was added to each sample and after incubation for 15 min at 37 °C in a thermal cycler, 100 μl of the sample was analysed on a MAGPIX device (Luminex). The analysis was performed at 37 °C. The protocol included a sample wash in the MAGPIX device and the minimum bead count was 50 microspheres of each region.

A negative control and a positive control for the reaction were included in each assay, except for the SNP assay in which only a negative control was included, since the wild-type allele acted as a positive control for the reaction. The negative control was a no template control (NTC) for which the DNA template was replaced by nuclease-free distilled water (Thermo Fisher Scientific) in the multiplex oligonucleotide ligation reaction. For the positive control DNA template, a single colony of each of five different isolates (i.e., samples S0001, S0002, S0024, S0025 and S0050 in Data set S1) was mixed in one tube with 50 μl sterile de-ionised water, which was treated as described in the section DNA isolation. Other isolates, separate or mixed, can be used as positive control for the reaction, as long as the performance of the reaction is verified for all markers in the MOL-PCR assay.

### PCR amplicon sequencing

For confirmation of the MOL-PCR results, PCR amplicons were sequenced on an ABI 3130xl Genetic Analyzer (Applied Biosystems). If primers were not available in literature, they were designed with Visual OMP (version 7.6.58.0; DNA Software). The amplicons were obtained by PCR as described above for the PCR screening with primers listed in Table S1 and were cleaned up before sequencing with ExoSAP-IT (Affymetrix) according to the manufacturer’s protocol. Sequence alignments were made with ClustalW in MegAlign (version 10.0.1 (3), 419; DNASTAR).

### Specificity of the internal positive control markers

The specificity of the internal positive control markers was tested by performing a MOL-PCR reaction with these probes on bacteria that are unrelated and closely related to *Salmonella* and on *Salmonella* isolates of other serovars than Typhimurium, but which are common in Belgium (Table S5) (Bertrand et al. 2014). All isolates used in this part of the development of the MOL-PCR assay were available in our laboratory as purified DNA (Barbau-Piednoir et al. 2013).

### Stability study

The in vitro stability of the selected molecular markers was evaluated in 31 *S*. Typhimurium and *S*. 1,4,[5],12:i:- isolates (indicated in Data set S1 in column ‘Stability_experiment’ with ‘1’ if included) with a common phage type in Belgium as follows: for each isolate, a single colony from a culture grown overnight on LB agar (Merck Millipore) at 37 °C was inoculated into 5 ml LB broth (Thermo Fisher Scientific) and incubated overnight at 37 °C without shaking. Next, a series of 50 passages at a rate of two passages per day was performed by inoculating 20 μl of culture into 5 ml fresh LB broth and incubating at 37 °C without shaking. Glycerol (25 % *v*/*v*) stocks (−80 °C) were made before each 5th passage. DNA was isolated after the 50th passage as described above.

### Data analysis

The output of the MAGPIX device includes the median fluorescence intensity (MFI) value for each marker in a comma-separated values file. These MFI values were read into R software (version 3.1.2) (R Core Team 2014). Signal-to-noise ratios (SN) were calculated by dividing the MFI of the sample by the corresponding MFI of the NTC (Eq. 1).

1 $$ S{N}_{\mathrm{sampl}{\mathrm{e}}_{\mathrm{marker}\kern0.5em \mathrm{a}}}=\frac{MF{I}_{{\mathrm{sampl}\mathrm{e}}_{\mathrm{marker}\kern0.5em \mathrm{a}}}}{MF{I}_{{\mathrm{NTC}}_{\mathrm{marker}\kern0.5em \mathrm{a}}}} $$

For the analysis of SNP markers, a SNP allele call was calculated by dividing the signal-to-noise ratio of the SNP marker by the sum of the signal-to-noise ratio of the SNP marker and the signal-to-noise ratio of its corresponding wild-type marker (Eq. 2). Analogously, a wild-type allele call was calculated by dividing the signal-to-noise ratio of the wild-type marker by the sum of the signal-to-noise ratio of the wild-type markers and the signal-to-noise ratio of its corresponding SNP marker (Eq. 3)

2 $$ Allele\kern0.5em  call\_SN{P}_{\mathrm{sample}\kern0.5em {\mathrm{x}}_{\mathrm{SNP}\kern0.5em \mathrm{a}}}=\frac{S{N}_{\mathrm{sample}\kern0.5em {\mathrm{x}}_{\mathrm{SNP}\kern0.5em \mathrm{a}}}}{S{N}_{\mathrm{sample}\kern0.5em {\mathrm{x}}_{\mathrm{SNP}\kern0.5em \mathrm{a}}}+S{N}_{\mathrm{sample}\kern0.5em {\mathrm{x}}_{\mathrm{WT}\kern0.5em \mathrm{a}}}} $$

3 $$ Allele\kern0.5em  call\_W{T}_{\mathrm{sample}\kern0.5em {\mathrm{x}}_{\mathrm{WT}\kern0.5em \mathrm{a}}}=\frac{S{N}_{\mathrm{sample}\kern0.5em {\mathrm{x}}_{\mathrm{WT}\kern0.5em \mathrm{a}}}}{S{N}_{\mathrm{sample}\kern0.5em {\mathrm{x}}_{\mathrm{WT}\kern0.5em \mathrm{a}}}+S{N}_{\mathrm{sample}\kern0.5em {\mathrm{x}}_{\mathrm{SNP}\kern0.5em \mathrm{a}}}} $$

### Data interpretation

During the development of the MOL-PCR assay, a universal cut-off value of 3 on the signal-to-noise ratio was used to determine the positive samples for markers that discriminate through presence or absence. For future application of the MOL-PCR as a routine subtyping assay, the cut-off values were refined for each of these markers after validation of the method on 519 *S*. Typhimurium and *S*. 1,4,[5],12:i:-. Hereto, the average of the maximum signal-to-noise ratio of the negative samples and the minimum signal-to-noise ratio of the positive samples was calculated (Tables S6 and S7). These average cut-off values were rounded to the nearest integer if the mean was greater than or equal to 3.75, to 3.5 if the mean was greater than or equal to 3.25 and smaller than 3.75 and to 3 if the mean was smaller than 3.25. For the SNP markers, the cut-off was set to 0.6 on the allele call, i.e., if the SNP allele call is greater than 0.6, then the SNP allele is assigned to the sample or if the wild-type allele call is greater than 0.6, then the wild-type allele is assigned to the sample. For the internal positive control markers, the cut-off value for the signal-to-noise ratio was calculated by rounding down the minimum signal-to-noise ratio of the positive samples to the nearest integer.

For the SNP markers, an additional cut-off was calculated on the MFI values to determine if the SNP locus was present, i.e., if the probes could hybridise to the SNP locus and subsequently be ligated and amplified. Hereto, the mean was determined between the maximum MFI of the negative samples for the allele and the minimum of the positive samples for the allele (Table S8). This mean was rounded to the nearest multiple of 100 if the mean was greater than or equal to 375 and to the nearest multiple of 50 if the mean was smaller than 375.

For the interpretation of multiplex data, each marker that discriminates through presence or absence and each SNP marker is assigned a unique prime number. If the marker is present in the sample, i.e., the signal-to-noise ratio or the SNP allele call is higher than the cut-off value, the sample receives the prime number of that marker. Otherwise, if the marker is absent, the sample receives ‘1’, which is the neutral element in the multiplication, for that marker. As such, the Gödel Prime Product (GPP) (Van den Bulcke et al. 2008, 2010) can be calculated as the product of all assigned prime numbers or ‘1’. Due to the nature of prime numbers, this GPP is a mathematical barcode (Gödel 1931) for the sample so that a large number of results can be assigned to a unique, arbitrary number, thereby simplifying data analysis on bacterial populations. An advantage of the GPP is that through factorisation of the GPP into its dividers, all discriminative markers present in the sample can be identified. Likewise, if the GPP is divided by the prime number of a specific marker, presence or absence of that marker will be indicated by, respectively, an integer or non-integer outcome of the division.

As the subtyping assay combines three MOL-PCRs, a MOL-PCR profile consists of three GPPs, i.e., GPP_MOL-PCR_1_–GPP_MOL-PCR_2_–GPP_MOL-PCR_SNP_. Unique prime numbers were assigned to each discriminative marker within each separate MOL-PCR (Tables S6, S7 and S8). To keep the GPPs as small as possible, the markers that were present in most samples received the lowest prime number; the markers that were present in the least amount of samples received the greatest prime number. As the GPPs may still result in a large number, an in-house code was assigned by ordering the GPPs, separately for each MOL-PCR, from the smallest to largest and numbering the GPPs starting from 1 (Tables S9, S10 and S11). An example of such an in-house code is 16-12-8.

**Fig. 1:**
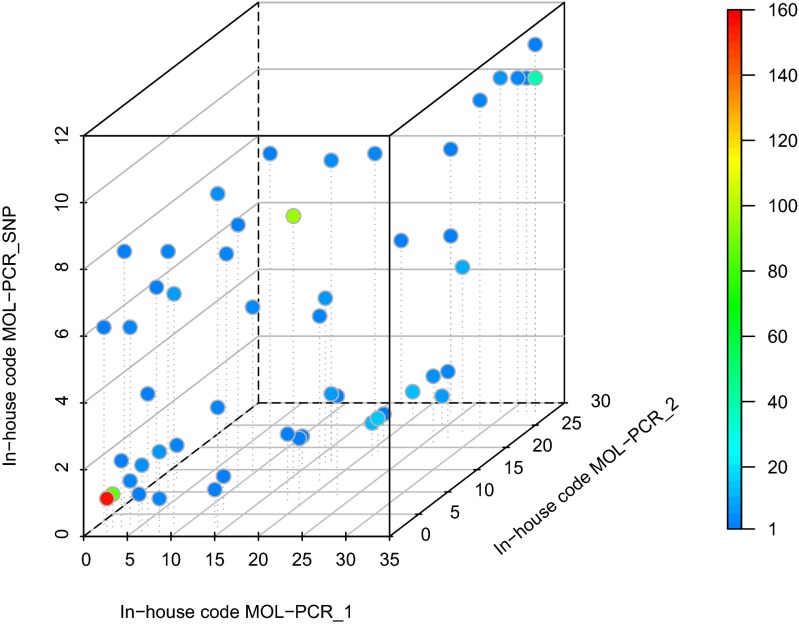
Visualisation of the 51 different MOL-PCR profiles (represented by 51 points) observed in the validation panel of 519 *S*. Typhimurium and *S*. 1,4,[5],12:i:- isolates with indication of the number of isolates in each profile, using a colour code. The colour code is automatically adapted to the number of isolates included in the data analysis with the R-application

Since a MOL-PCR profile consists of 3 numbers, the profiles were visualised in a 3-dimensional scatterplot in which the number of isolates in each MOL-PCR profile was indicated with a colour code. This type of visualisation may be informative for outbreak detection and can be realised in R software with the package scatterplot3d (version 0.3-35) (Ligges and Mächler 2003). As GPPs may be high numbers, the in-house code was used for each of the 3 axes in the scatterplot. An example of a MOL-PCR profile is 3.91 × 10^21^-1.11 × 10^13^-4199, which is in-house coded as MOL-PCR profile 33-28-10. The 3-dimensional scatterplot is then generated as follows: the numeral for GPP_MOL-PCR_1_, 3.91 × 10^21^, is in our example encoded as 33 and is plotted on the x-axis, the numeral for GPP_MOL-PCR_2_, 1.11 × 10^13^, is in our example encoded as 28 and is plotted on the y-axis, and the numeral for GPP_MOL-PCR_SNP_, 4199, is in our example encoded as 10 and is plotted against the z-axis so that finally, MOL-PCR profile 33-28-10 is represented as a point in the 3-dimensional scatterplot. The colour of a point indicates how many of the 519 isolates in the validation panel have that specific MOL-PCR profile. In our example, the MOL-PCR profile 33-28-10 is represented in Fig. 1 as the blue-green point in the top right corner, which indicates that about 40 isolates of our validation panel had MOL-PCR profile 3.91 × 10^21^-1.11 × 10^13^-4199, which was in-house coded as 33-28-10 (to be precise, 38 of the 519 isolates in our validation panel had this MOL-PCR profile).

An R-application for data analysis and interpretation, created with the package Shiny (version 0.11.1) (Chang et al. 2015) and which takes the MAGPIX output files as input, is available upon request.

### Discriminatory power

Discriminatory power was calculated as the average probability that two unrelated strains randomly sampled in the population are assigned a different type using Simpson’s index of diversity (Hunter and Gaston 1988).

## Results

### Assay design

MOL-PCR consists of three main steps: firstly, a multiplex oligonucleotide ligation of specific probes for detection of the molecular markers, secondly, a PCR for signal amplification and finally, the hybridisation to MagPlex-TAG microspheres and read-out on a Luminex device.

In the multiplex oligonucleotide ligation reaction, a different probe pair is included for each marker in the assay. If both probes of such a probe pair anneal adjacent to each other on the genomic DNA of the bacterial isolate to be tested, they are ligated by a thermostable DNA ligase so that various single-stranded DNA molecules are created that serve as a template in the subsequent singleplex PCR with a universal primer pair, i.e., T7 and T3. The T3 primer is 5′ biotinylated for read-out on a Luminex device. The third step starts with hybridisation of the PCR products to MagPlex-TAG microspheres, through a TAG that is integrated in the marker-specific probes and that is complementary to the anti-TAG covalently coupled to the surface of the microsphere. For each marker in the assay, a different TAG and a different MagPlex-TAG microsphere were used. Microspheres with a different anti-TAG have a different red colour code, which allows them to be identified by measurement of the red colour. After incubation with SAPE, a Luminex device will identify the microsphere through its red colour, and thus the marker, and measures the fluorescence signal of the SAPE to detect whether a PCR product has hybridised to the anti-TAG coupled to the microsphere.

A total of 70 potentially discriminative markers, including all 30 markers from the prophage subtyping assay of Fang et al. (2012), were selected from literature. The selection consisted of 32 genes of prophages Fels-1, Fels-2, Gifsy-1, Gifsy-2, P22, SopEφ, SLP281, ST64B, ST64T, ST104 and ST104B (Drahovská et al. 2007; Fang et al. 2012; Mikasová et al. 2005; Ross and Heuzenroeder 2005; Rychlík et al. 2008), 16 AFLP fragments (Fang et al. 2012; Lan et al. 2007), the left and right junction of SGI1 (Boyd et al. 2000; Rychlík et al. 2008), the allantoinase gene *allB* (Rychlík et al. 2008), MLVA locus STTR10 (Lindstedt et al. 2004), 12 SNPs (Pang et al. 2012), 5 antibiotic resistance genes encoded in SGI1 for resistance to ampicillin, chloramphenicol/florfenicol, streptomycin/spectinomycin, sulfonamides and tetracycline (Boyd et al. 2002; Ng et al. 1999), and the phase 2 flagellar gene *fljB* for identification of *S*. 1,4,[5],12:i:- (EFSA Panel on Biological Hazards (BIOHAZ) 2010).

### Selection of molecular markers

PCR screening was performed on the prophage genes, AFLP fragments, the left and right junction of SGI1 and the gene *allB*. Those markers which showed variation in at least 2 of the 30 tested isolates, and thus have discriminatory power, were selected for the MOL-PCR assay development. This criterion resulted in the rejection of 15 prophage genes and 7 AFLP fragments, of which, respectively, 6 and 5 markers were included in the prophage subtyping assay of Fang et al. (2012). However, although all 30 isolates in the PCR screening were negative for AFLP fragment markers SAL-36 (Fang et al. 2012), SAL-40 (Lan et al. 2007) and SAL-43 (Fang et al. 2012), these markers were not excluded since they showed variation among 8 DT1 *S*. Typhimurium isolates (S0031, S0032, S0036, S0041, S0042, S0043, S0049 and S0050 in Data set S1), which were screened earlier to determine a positive control for the PCR with these markers.

For each SNP marker, PCR amplicons were sequenced of at least one isolate with the SNP allele and one isolate with the wild-type allele. PCR amplicons were also sequenced for confirmation of MOL-PCR results that did not comply with previous results of the PCR screening. The sequences of all PCR amplicons confirmed the MOL-PCR assay results. For unpredicted negative MOL-PCR results (SAL-20, SAL-38, SAL-47, SAL-58 and SAL-71), mismatches were observed in the alignment of the PCR amplicon sequence and the target-specific sequence of the upstream and downstream probes, which prevented adequate annealing of the probes and explained why no ligation occurred. Polymorphisms in the binding site of primer SAL-10-F may explain the negative results for SAL-10 in the PCR screening for isolates S0007, S0023, S0025 and S0030 while positive results for SAL-10 in the MOL-PCR were seen for these isolates. Indeed, the target-specific sequence of the upstream and downstream probes of marker SAL-10 (SAL-10-U and SAL-10-D) aligned perfectly with the PCR amplicons generated for these isolates with primers SAL-10-R (same position as SAL-10-U) and SAL-10-F-nested, which is located downstream of SAL-10-F. Primer SAL-10-F was used in the PCR screening while SAL-10-F-nested was only used for PCR amplicon sequencing.

During the development of the MOL-PCR assay for subtyping, SNP SAL-57 was also eliminated, since no positive isolate could be identified and high background MFI values, as measured through the NTC, were obtained, even after redesigning of the probes.

As internal positive control markers, invasive gene *invA* (Barbau-Piednoir et al. 2013) and a SNP in the β subunit of RNA polymerase encoding gene *rpoB* (Hernández Guijarro et al. 2012) were selected. These markers target, respectively, all *Salmonella* species and *S*. Typhimurium and its monophasic variant *S*. 1,4,[5],12:i:-. To verify the specificity of markers *invA* and *rpoB*, the MOL-PCR assay was performed on isolates of species that are unrelated or closely related to *Salmonella* and on isolates of other serovars of *S. enterica* subsp. *enterica*. The results, summarised in Table S5, confirmed the specificity of the internal positive control makers.

As such, a total of 50 discriminative markers and 2 internal positive control markers were nominated for MOL-PCR assay development and these markers were distributed over 3 MOL-PCRs as indicated in Table 1. Twenty-one out of the 50 discriminative markers were also included in the subtyping assay of Fang et al. (2012).

### Stability study

The in vitro stability of the markers in the subtyping assay was examined by comparing the MOL-PCR profiles of 31 *S*. Typhimurium and *S*. 1,4,[5],12:i:- (indicated in Data set S1 in column Stability_experiment with ‘1’ if included) before and after an experiment of 50 serial passages in LB broth. No changes were observed in the MOL-PCR profiles of all 31 isolates before and after the 50 serial passages.

### Validation of the MOL-PCR assay for subtyping

For validation of the MOL-PCR assay for subtyping, the method was performed twice in independent assays on a collection of 519 *S*. Typhimurium and *S*. 1,4,[5],12:i:- isolates with a known phage type and MLVA profile, referred to as the validation panel (S0001-S0519 in Data set S1) and on 13 isolates related to 2 different outbreaks (S0520-S0532 in Data set S1). Each isolate was assigned the same MOL-PCR profile in both independent assays.

In the validation panel, 51 different MOL-PCR profiles were observed, which are presented in Fig. 1 and in Data set S1. The most common MOL-PCR profiles are profiles 15-1-1 (in-house coded as 2-1-1), 255255-8843835-1155 (16-12-8), 15-3-1 (2-2-1) and 3.91 × 10^21^-1.11 × 10^13^-4199 (33-28-10) which were observed for, respectively, 157 (30.3 % of the isolates in the validation panel), 97 (18.7 %), 86 (16.6 %) and 38 (7.3 %) isolates. All other profiles were detected in less than 15 isolates. The 341 *S*. Typhimurium isolates in the validation panel were grouped into 44 different MOL-PCR profiles and the 178 *S*. 1,4,[5],12:i:- into 7 different MOL-PCR profiles. According to the MOL-PCR results, the *fljB* gene could not be detected in all *S*. 1,4,[5],12:i:- isolates with the probe pair of marker SAL-73.

Markers SAL-50 and SAL-51 for detection of the left and right junction of SGI1 were observed in isolates with phage types DT104, U302, DT12, DT120 and DT110, of which, to our knowledge, all but DT110 have already been reported in literature (Boyd et al. 2002; Carattoli et al. 2002; Lawson et al. 2002).

The discriminatory power was calculated as Simpson’s index of diversity (*D*) on the 519 isolates in the validation panel and was 0.84 for the MOL-PCR assay, 0.84 for phage typing and 0.98 for MLVA.

Epidemiological concordance was evaluated by testing isolates related to two different outbreaks with the MOL-PCR assay. The first outbreak included isolates S0520 up to S0524 with S0525 as out-group; the second outbreak consisted of isolates S0526 up to S0530 with isolates S0531 and S0532 as out-group. The developed subtyping assay assigned identical MOL-PCR profiles to the outbreak isolates and separated them from their out-group isolates (Table 2).

**Table 2:** Subtyping data of *S*. Typhimurium and *S*. 1,4,[5],12:i:- isolates related to two different outbreaks

Sample	Serovar	MOL-PCR profile	MOL-PCR in-house code	Phage type	MLVA profile
S0520^a^	Typhimurium	1.91 × 10^7^-3.26 × 10^10^-1	21-19-1	DT195	3-12-10-NA-311
S0521^a^	Typhimurium	1.91 × 10^7^-3.26 × 10^10^-1	21-19-1	DT195	3-12-10-NA-311
S0522^a^	Typhimurium	1.91 × 10^7^-3.26 × 10^10^-1	21-19-1	DT195	3-12-10-NA-311
S0523^a^	Typhimurium	1.91 × 10^7^-3.26 × 10^10^-1	21-19-1	DT195	3-12-10-NA-311
S0524^a^	Typhimurium	1.91 × 10^7^-3.26 × 10^10^-1	21-19-1	DT195	3-12-10-NA-311
S0525^b^	Typhimurium	15-3-1	2-2-1	DT120	3-15-5-NA-211
S0526^c^	1,4,[5],12:i:-	15-1-1	2-1-1	DT138	3-13-11-NA-211
S0527^c^	1,4,[5],12:i:-	15-1-1	2-1-1	DT138	3-13-11-NA-211
S0528^c^	1,4,[5],12:i:-	15-1-1	2-1-1	DT138	3-13-11-NA-211
S0529^c^	1,4,[5],12:i:-	15-1-1	2-1-1	DT138	3-13-11-NA-211
S0530^c^	1,4,[5],12:i:-	15-1-1	2-1-1	DT138	3-13-11-NA-211
S0531^d^	Typhimurium	15-3-1	2-2-1	RDNC	3-14-11-NA-211
S0532^d^	Typhimurium	1.21 × 10^18^-2.58 × 10^11^-4199	30-23-10	DT104	3-14-18-14-311

*MLVA* multiple-locus variable-number of tandem repeats analysis, *MOL-PCR* multiplex oligonucleotide ligation-PCR, *RDNC* reacts-but-does-not-conform

^a^Isolates of the first outbreak

^b^Out-group isolate of the first outbreak

^c^Isolates of the second outbreak

^d^Out-group isolates of the second outbreak

### PFGE results

To examine if the isolates of the 3 most observed MOL-PCR profiles, i.e., 15-1-1, 255255-8843835-1155 and 15-3-1, in the validation panel could be further discriminated, PFGE was performed on a total of 53 isolates. Two clusters could be observed (Fig. S1). Cluster A grouped all isolates of MOL-PCR profiles 15-1-1 and 15-3-1, which differ only by marker SAL-73, i.e., *fljB*. Cluster B included all isolates of MOL-PCR profile 255255-8843835-1155. Cluster A was divided into 16 subgroups of which 2 subgroups comprised isolates of both MOL-PCR profiles 15-1-1 and 15-3-1. One subgroup in cluster B comprised 13 of the 17 isolates in this cluster while the other 3 subgroups consisted of only 1 or 2 isolates.

This dissimilarity in variation between cluster A and B for PFGE patterns is in agreement with the difference in number of distinct MLVA profiles in cluster A and B, 18 and 7 MLVA profiles, respectively, but contrasts with phage typing results, as in each cluster, 12 distinct phage types were identified. While PFGE could further divide isolates with the same MOL-PCR profile into separate subgroups, also MOL-PCR could make a distinction between isolates with the same PFGE pattern in 2 subgroups of cluster A. Similarly, isolates with the same MLVA profile (e.g., 3-12-9-NA-211) were assembled into distinct subgroups according to their PFGE patterns and are associated with different MOL-PCR profiles and phage types. Likewise, isolates with the same phage type are spread over clusters A and B with different PFGE patterns, MLVA and MOL-PCR profiles.

### Selection of most discriminative markers

It was observed that there were nine groups of two or three markers which were always present or absent together in the isolates of the validation panel: (1) SAL-10/SAL-23/SAL-42, (2) SAL-11/SAL-15, (3) SAL-16/SAL-27, (4) SAL-56/SAL-65, (5) SAL-49/SAL-61/SAL-63, (6) SAL-21/ SAL-33, (7) SAL-50/SAL-51, (8) SAL-37/SAL-38 and (9) SAL-36/SAL-40/SAL-43.

For each marker, Simpson’s index of diversity was calculated. The most discriminative markers were SAL-10/SAL-23/SAL-42, SAL-11/SAL-15, SAL-18, SAL-73 and SAL-16/SAL-27, thus 7 prophage gene markers, 1 AFLP marker and 1 SNP marker. The 51 observed MOL-PCR profiles could be reconstructed with a selection of 17 markers: SAL-11, SAL-16, SAL-18, SAL-23, SAL-26, SAL-29, SAL-35, SAL-36, SAL-53, SAL-55, SAL-62, SAL-66, SAL-67, SAL-70, SAL-71, SAL-73 and SAL-74. Hence, these markers encompass 8 prophage genes, 3 antibiotic resistance genes, 2 AFLP fragments, 2 SNPs, MLVA locus STTR10 and *fljB*.

## Discussion

In our attempt to design a complementary subtyping method for *S*. Typhimurium and its monophasic variant *S*. 1,4,[5],12:i:-, we have developed a MOL-PCR method for subtyping which combines different types of molecular markers in a high-throughput multiplex assay. We screen 52 molecular markers in 3 multiplex ligation assays, thereby avoiding the issues associated with multiplex PCR assays. Starting from a single colony, subtyping results are delivered within 8 h, which makes the MOL-PCR assay a convenient subtyping method for outbreak investigations, for which rapidity is of crucial importance. A similar turn-around-time is currently more difficult to be obtained with WGS. Another important aspect for outbreak investigations and for long-term surveillance studies is the stability of the assessed markers, which is not the case for all markers in MLVA (Dimovski et al. 2014) but which was demonstrated for our MOL-PCR assay by the results of a serial passage experiment. The data analysis and interpretation are objective and computerised, in contrast to that of phage typing. The data analysis results in a MOL-PCR profile consisting of 3 numerals, which can be easily compared between different laboratories (which is more difficult for PFGE) and straightforwardly stored in an electronic database, so that the developed subtyping method is suitable for use in an international surveillance network. Additionally, the presented visualisation as a 3-dimensional scatterplot is a flexible tool for outbreak detection when used with a limited number of isolates or for surveillance when used for a large collection of isolates, as the colour scheme will adapt itself to the number of isolates included in the data analysis. This objective data analysis is easily done by a non-bioinformatics expert, which might not be the case for WGS data analysis.

Besides rapidity, both Struelens et al. (1996) and van Belkum et al. (2007) propose flexibility, accessibility, cost and ease of use as convenience criteria for microbiological epidemiologic typing methods. The MOL-PCR assay is flexible in the sense that the technology of the MOL-PCR assay can be applied for (sub)typing of other pathogens. Nevertheless, for other pathogens, a different set of probes will have to be developed, as is also the case for e.g., MLVA and MLST.

The accessibility criterion deals with the availability of reagents and equipment and with the required skills for the method. A ligation reaction requires the same type of reagents, equipment and skills as a regular PCR, which is a generally used laboratory technique. Also for the hybridisation of the MOL-PCR products to MagPlex-TAG microspheres and incubation with SAPE, no special skills or equipment are necessary and required reagents are commonly available. The MOL-PCR assay was developed on a MAGPIX system, which stands at the lower end of the Luminex portfolio regarding cost and skills for use and maintenance of the system and which is feasible for a routine laboratory. The reagents and consumables cost for subtyping 1 isolate with the designed MOL-PCR method is lower than 10 euros, if the 3 MOL-PCR assays are combined on a 96-well plate so that 29 isolates are subtyped in 1 run.

The MOL-PCR method is designed for processing 96-well plates, which takes about 3.5 h hands-on time and requires no high-level technical skills. Analysis and interpretation of the results is straightforward by using the GPP and an available R-script and is thus not dependent on specialised commercial software. As such, this subtyping method scored well on the ease of use criterion.

In addition to stability of the assessed markers and suitability for computerised analysis and storage of results, both Struelens et al. (1996) and van Belkum et al. (2007) propose reproducibility, epidemiological concordance, discriminatory power (*D*) and typeability (*T*) as performance criteria for microbiological epidemiologic typing methods. As test population for the typing method, both authors recommend a large collection (*n* > 100) and Struelens et al. (1996) refine as ‘Large size collections of unrelated strains (*n* > 100), not selected on the basis of type characteristics, are recommended for the unbiased and precise comparison of the *T* and *D* values of different typing systems’. Our validation panel of 519 *S*. Typhimurium and *S*. 1,4,[5],12:i:- isolates complied with this recommendation.

A reproducible method is able to assign the same type to an isolate that was tested multiple times and in an independent manner. The reproducibility of the MOL-PCR assay was proven by an independent repeat of the subtyping method, in which all isolates of the validation panel and of the 2 outbreaks received the same MOL-PCR profile as in the initial experiment. In contrast to PFGE, the molecular techniques used in the MOL-PCR assay, i.e., ligation, PCR and hybridisation to microspheres, can be standardised without great effort, so that results may also be reproducible between different laboratories.

In the experiment for the assessment of the epidemiological concordance, identical MOL-PCR profiles were observed for outbreak isolates, which were clearly distinguished from their out-group isolates. These results concurred with results of phage typing and MLVA, although separation of out-group isolate S0531 (Table 2) from the respective outbreak isolates would be difficult with only MLVA data since its MLVA profile was different from the MLVA profile of the outbreak isolates in only one repeat at an instable locus (Dimovski et al. 2014).

The instability of 3 MLVA loci may also explain the higher value for Simpson’s index of diversity (*D*) for MLVA compared to the MOL-PCR assay, as large numbers of MLVA profiles are produced as a result of these rapidly evolving loci, which make MLVA less suitable for investigation of long-lasting outbreaks and long-term surveillance. According to Simpson’s index of diversity, the MOL-PCR method has the same discriminatory power as phage typing. However, whereas phage typing produces NT and RDNC results, a 100 % typability was observed in the MOL-PCR assay since all of the 519 isolates of the validation panel were assigned a MOL-PCR profile and could thus be subtyped by the assay. Compared to the subtyping method described by Fang et al. (2012), based on multiplex PCR with detection through a direct hybridisation assay, the developed MOL-PCR assay provides an increased discrimination since the ligation reaction only occurs under strict conditions: the upstream probe has to hybridise exactly adjacent to the downstream probe and a strict complementarity is compulsory for the base pairs flanking the ligation site. Even so, this more stringent discrimination was not reflected in the discriminatory power as calculated by Simpson’s index of diversity, which was 0.84 for the MOL-PCR method compared to 0.95 for the multiplex PCR-based method (Fang et al. 2012). This might be explained by the dissimilar test panels used for evaluation of both subtyping methods. Our validation panel of 519 *S*. Typhimurium and *S*. 1,4,[5],12:i:- isolates included 33 distinct phage types, whereas Fang et al. (2012) tested a selected panel of 438 *S*. Typhimurium representing 58 phage types and thus calculated Simpson’s index of diversity on a smaller collection with a higher variation. Moreover, simple adaptations to the collection of isolates tested may have significant effects on Simpson’s index of diversity, e.g. if half of the isolates with the 3 most frequently observed MOL-PCR profiles is left out of the validation panel, which would then be reduced to 350 isolates, still above the recommended size of >100 isolates, Simpson’s index of diversity would increase to 0.95 for the MOL-PCR assay and would thus comply with the acceptable discriminatory power for a “more or less ‘ideal’” typing system (van Belkum et al. 2007). As Simpson’s index of diversity is very much dependent on the collection of isolates tested, a more strict definition of such a test panel might be required for evaluation of subtyping methods.

The MOL-PCR assay with its visualisation tool (Fig. 1) and coupled with insightful epidemiological data, offers a user-friendly and rapid approach for outbreak investigations. If, however, a frequent MOL-PCR profile (i.e., 15-1-1, 255255-8843835-1155, 15-3-1 and 3.91 × 10^21^-1.11 × 10^13^-4199) would be obtained, the isolate might be further characterised by MLVA, PFGE or WGS. Nonetheless, due to the instability of three MLVA loci in *S*. Typhimurium (Boxrud 2010; Dimovski et al. 2014), it may not be clear how to handle isolates that differ in one of the instable loci (Friesema et al. 2012; Garvey et al. 2013; Kuhn et al. 2013; Paranthaman et al. 2013; Petersen et al. 2011). Therefore, for current routine laboratories, PFGE might be more appropriate. In our case, PFGE could further discriminate isolates with the same MOL-PCR profile, but at the other hand, different MOL-PCR profiles were obtained for isolates within the same PFGE cluster. This is even more an issue with MLVA profiles. This interwoven tangle of subtyping results illustrates the complications that are encountered when comparing different sets of subtyping data, which will only become more complicated when WGS data will be compared to historical subtyping results. Also, with WGS, agreements will have to be made within the community as to decide whether two isolates are identical or not, as the resolution is at the single nucleotide level and in vivo/in vitro mutations or sequencing errors might occur.

The discrimination for the most frequently observed MOL-PCR profiles can be increased by including more markers in the MOL-PCR assay. Such additional markers may be identified by WGS comparison of different isolates with the same commonly observed MOL-PCR profile. However, if more markers are included in the assay, this may lead to an increase of the cost and effort of the method. To avoid an expansion of the MOL-PCR assay, redundant markers which were present or absent together in the isolates of the validation panel could be removed. However, the markers that are redundant for our validation panel, could be critical for discrimination when applying the MOL-PCR assay to future collections of *S*. Typhimurium and *S*. 1,4,[5],12:i:- isolates. Therefore, it might be more appropriate to evaluate the redundancy of the molecular markers included in the method after routinely subtyping *S*. Typhimurium and its monophasic variant *S*. 1,4,[5],12:i:- with the MOL-PCR assay for a period of e.g., 3 years in multiple reference laboratories.

Ultimately, WGS might become the gold standard for subtyping of any pathogen, but during the time that not all routine laboratories have the resources and data analysis capabilities and agreements on interpretation for WGS of *S*. Typhimurium and its monophasic variant *S*. 1,4,[5],12:i:-, the developed MOL-PCR assay may be considered as an inexpensive complement of currently applied methods in routine subtyping with a readily accessible, computerised data analysis pipeline.

## Electronic supplementary material

ESM 1 (PDF 1337 kb)
