# Supplementary material for: A multiplex oligonucleotide ligation-PCR as a complementary tool for subtyping of Salmonella Typhimurium
Source: Appl Microbiol Biotechnol. 2015 Jul 25;99(19):8137–49. doi: 10.1007/s00253-015-6831-7 (PMC4561068; doi:10.1007/s00253-015-6831-7)
Supplement: Supplementary file 1 — (PDF 1337 kb) [file 253_2015_6831_MOESM1_ESM.pdf]

Applied Microbiology and Biotechnology

A multiplex oligonucleotide ligation-PCR as a complementary tool for subtyping of *Salmonella* Typhimurium

Véronique Wuyts[a,b,c], Wesley Mattheus[d], Nancy H.C. Roosens[c], Kathleen Marchal[a,b,e], Sophie Bertrand[d§], Sigrid C.J. De Keersmaecker[c§#]

[a]Department of Microbial and Molecular Systems, KU Leuven, Leuven, Belgium

[b]Department of Plant Biotechnology and Bioinformatics, Ghent University, Ghent, Belgium

[c]Platform Biotechnology and Molecular Biology, Scientific Institute of Public Health (WIV-ISP), Brussels, Belgium

[d]National Reference Centre for *Salmonella* and *Shigella*, Bacterial Diseases Division, Communicable and Infectious Diseases, Scientific Institute of Public Health (WIV-ISP), Brussels, Belgium

[e]Department of Information Technology, Ghent University, IMinds, Ghent, Belgium

[§]Equally contributed

[#]e-mail: sigrid.dekeersmaecker@wiv-isp.be, telephone: +32(0)26425257, fax: +32(0)26425292

Fig. S1 PFGE results of 53 isolates of the 3 most observed MOL-PCR profiles in the validation panel of 519 *S*. Typhimurium and *S*. 1,4,[5],12:i:- isolates. A horizontal line between two isolates indicates that these isolates were run on different gels (4 gels in total)

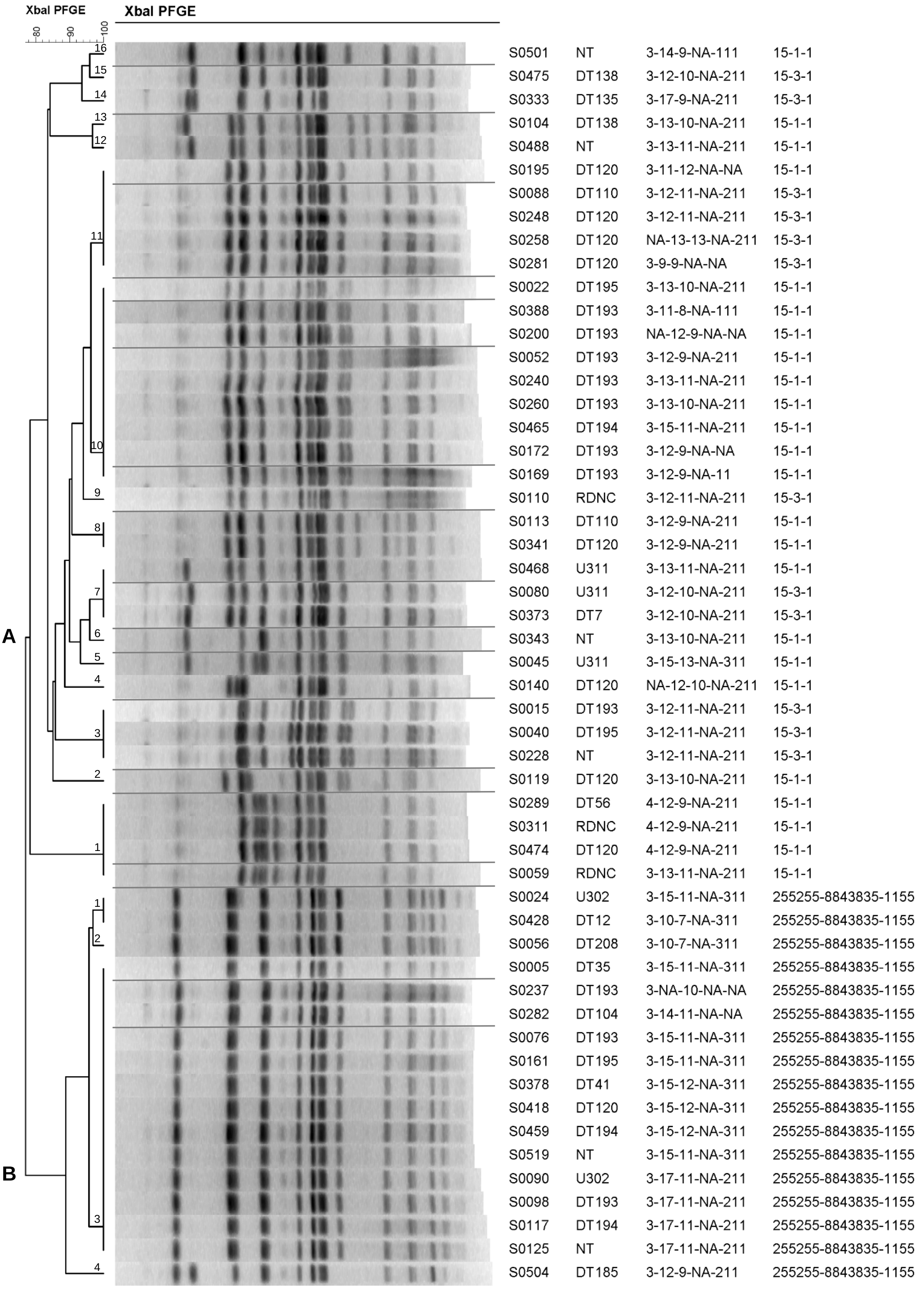

Table S1 Primer pairs used in the development of the MOL-PCR subtyping assay

Target	Targeted sequence	Primer	Sequence (5'→3')[a]	Annealing temp (°C)	Concn (nM)	Amplicon size (bp)	Reference
ST64B	*immC cl*-5'	SAL-1-F	ATGGTGGCCTTGTCGACGC	58	400	475	(Ross and Heuzenroeder 2005)
		SAL-1-R	GCTAACGTGAAGGATTTGTTCCG		400		(Ross and Heuzenroeder 2005)
ST64B	*immC cl*-3'	SAL-2-F	CCATTACCGGCGCTTGCAC	58	400	453	(Ross and Heuzenroeder 2005)
		SAL-2-R	TAACGTATAACCATGCGATTTCCG		400		(Ross and Heuzenroeder 2005)
ST64B	*immC cro*	SAL-3-F	GCGATATACGCAAAAGAAGGAGG	58	400	518	(Ross and Heuzenroeder 2005)
		SAL-3-R	TGGCTACTGAATGTGCCAGG		400		(Ross and Heuzenroeder 2005)
ST64B	*immC* put *c2*	SAL-4-F	GCTGGTACTGCAACGTGCC	58	400	568	(Ross and Heuzenroeder 2005)
		SAL-4-R	CGAATGACATGGACATAAAGTCC		400		(Ross and Heuzenroeder 2005)
ST64B	SB6	SAL-5-F	ACGACAAGCGCGTTGAGGC	58	400	280	(Fang et al. 2012)
		SAL-5-R	ACGGCATAACCACCATCTTCATC		400		(Fang et al. 2012)
ST64B	SB26	SAL-6-F	GACACCATCAATGTATGGATCGC	58	400	276	(Fang et al. 2012)
		SAL-6-R	GGAAGTAATGCTTTTCTGGAGGC		400		(Fang et al. 2012)
ST64B	SB28	SAL-7-F	CGTGGAAAGTGCAGTCAAGAGG	58	400	211	(Fang et al. 2012)
		SAL-7-R	CAAGGAAAAACCGGAGTTAAACAA		400		(Fang et al. 2012)
ST64B	SB37	SAL-8-F	TGGTAGTGAATTGGTTAGCTGCG	58	400	279	(Fang et al. 2012)
		SAL-8-R	GCAATAAAGGCAAGGTAGTCCTCA		400		(Fang et al. 2012)
ST64B	SB46	SAL-9-F	CATTGATGGTATCGAAGTTCGCC	58	400	490	(Ross and Heuzenroeder 2005)
		SAL-9-R	CCTGGAGTTTCTGGCACGC		400		(Ross and Heuzenroeder 2005)
ST64T/P22	g9-5'	SAL-10-F[b]	GATTCCTTGCATCTGGAGCAC	58	400	217	(Fang et al. 2012)
		SAL-10-R	GGCTGAGCAATCTGGACGTG		400		(Fang et al. 2012)
		SAL-10-F-nested[c]	GTTGTTTCTAACCCTCGTCC		400	171	This study
ST64T/P22	g9-3'	SAL-11-F	TACCGTAGAAGATTGCGCTGG	58	400	276	(Fang et al. 2012)
		SAL-11-R	AACCCTTCTTCTGCCAAATTAGC		400		(Fang et al. 2012)
ST64T/P22	g17	SAL-12-F	AGCGTTGCAGTAATCAGGTTTG	58	400	163	(Fang et al. 2012)
		SAL-12-R	AGGAAATATGAAATTACGTGTCTGG		400		(Fang et al. 2012)
ST64T/P22	*gtrA*	SAL-13-F	TTGCCGAGAAAGTGATAAGGGTAA	58	400	179	(Fang et al. 2012)
		SAL-13-R	TGTTATCGCCGTATCGTTCAGC		400		(Fang et al. 2012)

Table S1 (continued)

Target	Targeted sequence	Primer	Sequence (5'→3')[a]	Annealing temp (°C)	Concn (nM)	Amplicon size (bp)	Reference
ST64T/P22	*gtrB*	SAL-14-F	GGATCAGATGCGGCTATTTTG	58	400	242	(Fang et al. 2012)
		SAL-14-R	TTTCTCGGCAATTAGCCTGG		400		(Fang et al. 2012)
ST64T/P22	*gtrC*	SAL-15-F	GCATTAACACCTCTGACCACATCC	58	400	229	(Fang et al. 2012)
		SAL-15-R	GCTTACAATGCCATAAATGCACA		400		(Fang et al. 2012)
ST64T	*mnt*	SAL-16-F	TACAGGCGTAGGCTTTTCAAGTG	58	400	219	(Fang et al. 2012)
		SAL-16-R	CGGTGGTCTTATTGTGGATATAACC		400		(Fang et al. 2012)
ST64T/P22	*int*	SAL-18-F	CATTTCCTGCAATACCGAAATCGG	58	400	230	(Fang et al. 2012)
		SAL-18-R	AGCAAATCGGCAATCATCACAA		400		(Fang et al. 2012)
P22	*sieB*	SAL-20-F	CGATGAACAACTCATGGTGGC	58	400	227	(Fang et al. 2012)
		SAL-20-R	GGAATTAACAACACCGTTAAGCAC		400		(Fang et al. 2012)
ST64T/P22	*eac*	SAL-21-F	GATTGCATCCAGCTCTTTGTTGTA	58	400	144	(Fang et al. 2012)
		SAL-21-R	TTCGTTTGGGAAAAAGGATATGA		400		(Fang et al. 2012)
ST64T/P22	g8	SAL-23-F	TGCCAACGACAATGCAGGTC	58	400	210	(Fang et al. 2012)
		SAL-23-R	TGAGGWGGAAGGTCAGGGTTTAC		400		(Fang et al. 2012)
P22	g13	SAL-24-F	GCAAAGGAACAGGGCATCG	58	400	244	(Fang et al. 2012)
		SAL-24-R	TAGCAGCGAAGCGTTTGATTAG		400		(Fang et al. 2012)
ST104	g9	SAL-25-F	CGTGGGTAGACGATTTCACTG	58	400	187	(Drahovská et al. 2007)
		SAL-25-R	CGAAGTTGGTCTGCACAATG		400		(Drahovská et al. 2007)
ST104	g44	SAL-26-F	TGATGAGGACGGACGCTACAAC	58	400	218	(Fang et al. 2012)
		SAL-26-R	CAACGCCCCACACACCAGG		400		(Fang et al. 2012)
ST104	g61	SAL-27-F	TGCGCTACAGCAACAAAAACG	58	400	199	(Fang et al. 2012)
		SAL-27-R	CAAATGCGTTGCCAGTACTTCC		400		(Fang et al. 2012)
Gifsy-1	*gipA*	SAL-29-F	GGCAAGCTGTACATGGCAAAG	58	400	214	(Fang et al. 2012)
		SAL-29-R	CGGTATCGGTGACGAACAAATC		400		(Fang et al. 2012)
Gifsy-2	*gtgB*	SAL-30-F	TGCACGGGGAAAACTACTTC	58	400	436	(Drahovská et al. 2007; Mikasová et al. 2005)
		SAL-30-R	TGATGGGCTGAAACATCAAA		400		(Drahovská et al. 2007; Mikasová et al. 2005)
Fels-1	*nanH*	SAL-31-F	GTGAGCCGCGACAAGTTTAT	58	400	807	(Drahovská et al. 2007; Mikasová et al. 2005)
		SAL-31-R	TCTGGGGGAACAACCAAATA		400		(Drahovská et al. 2007; Mikasová et al. 2005)
SopEφ	*sopE*	SAL-32-F	GTGGAGTCGGCATAGCACACTC	58	400	191	(Fang et al. 2012)
		SAL-32-R	AAATGACGTTTTTACGCCAAGC		400		(Fang et al. 2012)
AFLP fragments	CA-1	SAL-33-F	AATTCATATCCTTTTTCCCAAACG	45	400	133	(Fang et al. 2012)
		SAL-33-R	TTAACGATCAACGAAATTCAATCC		400		(Fang et al. 2012)

Table S1 (continued)

Target	Targeted sequence	Primer	Sequence (5'→3')[a]	Annealing temp (°C)	Concn (nM)	Amplicon size (bp)	Reference
AFLP fragments	CA-2	SAL-34-F	ATGATGCTGCATATTCGATAAGGT	58	400	209	(Fang et al. 2012)
		SAL-34-R	ATTCAGGCTATCTGTGTATTGGTGA		400		(Fang et al. 2012)
AFLP fragments	CA-3	SAL-35-F	CGCCATCCCGATTGTTTATTG	58	400	148	(Fang et al. 2012)
		SAL-35-R	TGGCTGGCAGGGTCTGTTC		400		(Fang et al. 2012)
AFLP fragments	CA-7	SAL-36-F	AATTCATCGGGGTGATCAGCA	58	400	129	(Fang et al. 2012)
		SAL-36-R	TTGCCGGGAGTGATACGAGC		400		(Fang et al. 2012)
AFLP fragments	CA26.1	SAL-37-F	GCGGCGCGTATTTCGTGC	58	400	137	(Lan et al. 2007)
		SAL-37-R	GCAGAACTGGAAGCACGG		400		(Lan et al. 2007)
AFLP fragments	CA28.4	SAL-38-F	TGATGCACTAATTCGTCG	58	400	119	(Lan et al. 2007)
		SAL-38-R	AATTCAGCATTGGCCTGC		400		(Lan et al. 2007)
AFLP fragments	CG-1	SAL-39-F	GCAGCAGGGAACCGTCTTGA	58	400	164	(Lan et al. 2007)
		SAL-39-R	TCATTCCTGTATGCCTGAAGTTTG		400		(Fang et al. 2012)
AFLP fragments	CG-2	SAL-40-F	CTATATCACGCTGAACAGAC	58	400	81	(Lan et al. 2007)
		SAL-40-R	ATACCTGCTCTTTCTGCTCT		400		(Lan et al. 2007)
AFLP fragments	GC-1	SAL-48-F	TGGAAGAACAAGCAAACAAGATTC	58	400	164	(Fang et al. 2012)
		SAL-48-R	ATGATACGGCGTGCCACATC		400		(Fang et al. 2012)
Allantoinase	*allB*	SAL-49-F	TTTCGCGACGTTAATGACT	60	400	560	(Rychlík et al. 2008)
		SAL-49-R	TCAAACATGACGTCCATGC		400		(Rychlík et al. 2008)
S*almonella* genomic island 1 (SGI1)	Left junction	SAL-50-F	TTACCGGCGAGTTTACCTC	60	400	438	(Rychlík et al. 2008)
		SAL-50-R	TCTGCTTGTGTCTTTGGGT		400		(Rychlík et al. 2008)
S*almonella* genomic island 1 (SGI1)	Right junction	SAL-51-F	TGACGAGCTGAAGCGAATTG	60	400	515	(Boyd et al. 2000)
		SAL-51-R	AGCAAGTGTGCGTAATTTGG		400		(Boyd et al. 2000)
SLP281	SL1344_2641	SAL-52-F	GACTAAAGCGGGATACCGTA	60	400	425	(Rychlík et al. 2008)
		SAL-52-R	GGGCTGTCTCTTTCAGCAG		400		(Rychlík et al. 2008)
Gifsy-1	*artA*	SAL-53-F	TCTGGTTATGCAAGTGCTGT	60	400	263	(Rychlík et al. 2008)
		SAL-53-R	TCTGCACGGATTCTGTATCTA		400		(Rychlík et al. 2008)
Fels-2	STM2697	SAL-54-F	ACGAAAAAGCTCAGTAGTGC	60	400	394	(Rychlík et al. 2008)
		SAL-54-R	TAGTCTATAGCGCCGTTCTC		400		(Rychlík et al. 2008)
ST104B	*hldD* homolog	SAL-55-F	CGCAGTAGAGACATGGATGTA	60	400	280	(Rychlík et al. 2008)
		SAL-55-R	CTGGCGGTACAGCTTTATG		400		(Rychlík et al. 2008)
SNP TM81	STM0080	SAL-56-F	GCAGCAACACTATTCACC	55	200	741	(Hu et al. 2006)
		SAL-56-R	GCCCTTTTACCCCCTCTT		200		(Hu et al. 2006)
SNP A/A-327 & T/A-590A	*tktA*	SAL-57-F	TTACGGCTGGCGATTTT	48	500	536	(Hu et al. 2006)
		SAL-57-R	TTCTATGATGACAACGG		500		(Hu et al. 2006)
SNP TM3230_1	intergenic	SAL-58-F	CGAAGCGGAAGCAAACAG	48	200	597	(Hu et al. 2006)
		SAL-58-R	GTAATAACGGGGGATAGC		200		(Hu et al. 2006)

Table S1 (continued)

Target	Targeted sequence	Primer	Sequence (5'→3')[a]	Annealing temp (°C)	Concn (nM)	Amplicon size (bp)	Reference
SNP TM1231	STM1269	SAL-59-F	TGAAGAAGAAGCGAAAAA	55	200	808	(Hu et al. 2006)
		SAL-59-R	CCAAGGTAGAGCAGATAA		200		(Hu et al. 2006)
SNP TM973	STM1002	SAL-60-F	ATTTCCGCTGATGACTGT	55	200	732	(Hu et al. 2006)
		SAL-60-R	GCCACCTCTATTGATTTTA		200		(Hu et al. 2006)
SNP 1880	*otsA*	SAL-61-F	TCTCCGCATGACGCGAAATA	60	200	306	This study
		SAL-61-R	GCTGGACGCCGCTCTATTAT		200		This study
SNP 2199	*napF*	SAL-62-F	CCGGATAACGTGGGACGAAA	60	200	208	This study
		SAL-62-R	GCGTAGATTTCAAACGGGGC		200		This study
SNP 101	*araD*	SAL-63-F	GTCATTTTGCGAGTGCAGGG	60	200	215	This study
		SAL-63-R	GAGAGCGGTGAAGTCGTTGA		200		This study
SNP TM3124_1	intergenic	SAL-64-F	GAGGCGTAAAGCGACAG	55	200	776	(Hu et al. 2006)
		SAL-64-R	AATCCAGCGTAGGGTAG		200		(Hu et al. 2006)
SNP TM3275_2	intergenic	SAL-65-F	TTGAAGAGGCGTGTTGG	55	200	590	(Hu et al. 2006)
		SAL-65-R	GCTGAAAGGCGAAGAAT		200		(Hu et al. 2006)
Streptomycin/	*aadA2*	SAL-66-F	TGTTGGTTACTGTGGCCG	55	500	538	(Ng et al. 1999)
spectinomycin resistance		SAL-66-R	TGCTTAGCTTCAAGTAAGACG		500		(Boyd et al. 2002)
SNP 4213	*yjeF*	SAL-71-F	AGCCGGTCTGATTGAACAGG	60	200	616	This study
		SAL-71-R	ATCCGCATCCCACAGCATAG		200		This study
SNP TM2079	intergenic	SAL-72-F	TCAGAAGGAGGGAGCAA	55	200	701	(Hu et al. 2006)
		SAL-72-R	GCGAAGTAGAGTAATGG		200		(Hu et al. 2006)
MLVA locus STTR10	PSLT064	SAL-74-F	CGGGCGCGGCTGGAGTATTTG	60	200	[d]	(Lindstedt et al. 2004)
		SAL-74-R	GAAGGGGCCGGGCAGAGACAGC		200		(Lindstedt et al. 2004)

[a] W: A or T

[b] Used for initial PCR screening of the marker

[c] Used for sequencing of PCR amplicons

[d] Amplicon length depends on number of repeats (x) present in the locus: amplicon length = 311 + (6 × x)

Table S2 Probes used in MOL-PCR_1

Target	Targeted sequence	Probe	Probe pre-mix	Sequence (5’→3’)	Bead region	Accession no.	Reference of target-specific sequence
All *Salmonella species*	*invA*	invA-U	1.1	*TAATACGACTCACTATAGGG*gataagaaagtgaaatgtaaattgATAAACTTCATCGCACCGTCA	51	AE006468 (McClelland et al. 2001)	(Barbau-Piednoir et al. 2013)
		invA-D	1.1	P-AAGGAACCGTAAAGCTGGCTT*TCCCTTTAGTGAGGGTTAAT*			
Salmonella enterica subsp. *enterica* serovar Typhimurium	*rpoB*	rpoB-U	1.2	*TAATACGACTCACTATAGGG*gtaattgaattgaaagataagtgtTTTCTCAGCTGCACCGTAG**C**	18	AE006468 (McClelland et al. 2001)	(Hernández Guijarro et al. 2012)
		rpoB-D	1.2	P-CCTGGCGTCTTCTTTGACTCC*TCCCTTTAGTGAGGGTTAAT*			
ST64T/P22	g9-5’	SAL-10-U	-	*TAATACGACTCACTATAGGG*gtgtgttatttgtttgtaaagtatGGCTGAGCAATCTGGACGTG	19	AY052766 (Mmolawa et al. 2003)	(Fang et al. 2012)
		SAL-10-D	-	P-AGAGCCATCCTCATTTTCAA*TCCCTTTAGTGAGGGTTAAT*			
ST64T/P22	g9-3’	SAL-11-U	1.2	*TAATACGACTCACTATAGGG*gtaagagtattgaaattagtaagaAACCCTTCTTCTGCCAAATTAGC	66	AY052766 (Mmolawa et al. 2003)	(Fang et al. 2012)
		SAL-11-D	1.2	P-AACATTAATTCTAGAGGGGTCTACC*TCCCTTTAGTGAGGGTTAAT*			
ST64T	*mnt*	SAL-16-U	1.2	*TAATACGACTCACTATAGGG*tgtatatgttaatgagatgttgtaTACAGGCGTAGGCTTTTCAAGTG	37	AY052766 (Mmolawa et al. 2003)	(Fang et al. 2012)
		SAL-16-D	1.2	P- AATCAGCCACTATCTGCACT*TCCCTTTAGTGAGGGTTAAT*			
ST64T/P22	g8	SAL-23-U	1.2	*TAATACGACTCACTATAGGG*agtagaaagttgaaattgattatgTGCCAACGACAATGCAGGTC	12	NC_002371 (Pedulla et al. 2003)	(Fang et al. 2012)
		SAL-23-D	1.2	P-AGGAAGAGGGCTTTGAGATTG*TCCCTTTAGTGAGGGTTAAT*			
ST104	g9	SAL-25-U	-	*TAATACGACTCACTATAGGG*gaagatattgaaagaatttgatgtCGAAGTTGGTCTGCACAATG	55	AB102868 (Tanaka et al. 2004)	(Drahovská et al. 2007)
		SAL-25-D	-	P-CAGCAGGGCGTCCGTCGCTTC*TCCCTTTAGTGAGGGTTAAT*			
Gifsy-1	*gipA*	SAL-29-U	1.1	*TAATACGACTCACTATAGGG*tttaagtgagttatagaagtagtaGGCAAGCTGTACATGGCAAAG	29	AF246666 (Stanley et al. 2000)	(Mikasová et al. 2005)
		SAL-29-D	1.1	P-AACAAAATCCCCTTAGACG*TCCCTTTAGTGAGGGTTAAT*			
AFLP fragments	CA-1	SAL-33-U	-	*TAATACGACTCACTATAGGG*gttgagaattagaatttgataaagTTAACGATCAACGAAATTCAATCC	73	AF500153 (Hu et al. 2002)	(Fang et al. 2012)
		SAL-33-D	-	P-TAACCACAGCCGCTGAAAAGGT*TCCCTTTAGTGAGGGTTAAT*			
AFLP fragments	GA-1	SAL-45-U	1.2	*TAATACGACTCACTATAGGG*gttagttatgatgaatattgtgtaGCGTGGAATATCGTTGATGG	45	AF500166 (Hu et al. 2002)	(Lan et al. 2007)
		SAL-45-D	1.2	P-GTTTGAAGCGGTGTCGAA*TCCCTTTAGTGAGGGTTAAT*			

Table S2 (continued)

Target	Targeted sequence	Probe	Probe pre-mix	Sequence (5'→3')	Bead region	Accession no.	Reference of target-specific sequence
Allantoinase	*allB*	SAL-49-U	1.2	*TAATACGACTCACTATAGGG*agtaagtgttagatagtattgaatTTTCGCGACGTTAATGACT	38	AE006468 (McClelland et al. 2001)	(Rychlík et al. 2008)
		SAL-49-D	1.2	P-GGCAGTTTTACAAAGGCGCGC*TCCCTTTAGTGAGGGTTAAT*			
S*almonella* genomic island 1 (SGI1)	Left junction	SAL-50-U	1.1	*TAATACGACTCACTATAGGG*gtttgtgtttgtataagttgttaaTCTGCTTGTGTCTTTGGGT	53	AF261825 (Boyd et al. 2001)	(Rychlík et al. 2008)
		SAL-50-D	1.1	P-TCTCGTAGAGATAGAGTTCTAAAGG*TCCCTTTAGTGAGGGTTAAT*			
S*almonella* genomic island 1 (SGI1)	Right junction	SAL-51-U	1.1	*TAATACGACTCACTATAGGG*tagagaaagagagaattgtattaaTGACGAGCTGAAGCGAATTG	54	AF261825 (Boyd et al. 2001)	(Boyd et al. 2001)
		SAL-51-D	1.1	P-CAGGGCTGAACAGCTCAA*TCCCTTTAGTGAGGGTTAAT*			
Gifsy-1	*artA*	SAL-53-U	1.2	*TAATACGACTCACTATAGGG*aaataagaatagagagagaaagttTCTGGTTATGCAAGTGCTGT	43	HF937208 (Mather et al. 2013)	(Rychlík et al. 2008)
		SAL-53-D	1.2	P-TGATTTTGTATATCGTGTTGACTCGAG*TCCCTTTAGTGAGGGTTAAT*			
ST104B	*hldD* homolog	SAL-55-U	1.2	*TAATACGACTCACTATAGGG*aatgtaaagtaaagaaagtgatgaCGCAGTAGAGACATGGATGTA	44	HF937208 (Mather et al. 2013)	(Rychlík et al. 2008)
		SAL-55-D	1.2	P-CCAAGTTGCACAGCGAAC*TCCCTTTAGTGAGGGTTAAT*			
Chloramphenicol/ florfenicol resistance	*floR*	SAL-67-U	1.3	*TAATACGACTCACTATAGGG*ttgtgatagtagttagatatttgtGCGGAATATTCCGAGATCGGATTCAGC	39	AF261825 (Boyd et al. 2001)	This study
		SAL-67-D	1.3	P-TTTGCCTTCGCCACTGTCGCGC*TCCCTTTAGTGAGGGTTAAT*			
Tetracycline resistance	*tet*(G)	SAL-68-U	-	*TAATACGACTCACTATAGGG*gattgatatttgaatgtttgtttgGAACGGTTGGGTTTGGATTGTCG	22	AF261825 (Boyd et al. 2001)	This study
		SAL-68-D	-	P-GCGCGATCCTCTATTTAATATGTCTGCC*TCCCTTTAGTGAGGGTTAAT*			
Ampicillin resistance	*bla*$_{\text{PSE-1}}$	SAL-69-U	1.3	*TAATACGACTCACTATAGGG*gtgattgaatagtagattgtttaaCCCAATAGTACAGTCGAGATT	46	AF261825 (Boyd et al. 2001)	This study
		SAL-69-D	1.3	P-AAGAAAGCAGATCTTGTGACC*TCCCTTTAGTGAGGGTTAAT*			
Sulfonamide resistance	*sul1*	SAL-70-U	1.3	*TAATACGACTCACTATAGGG*gttgtaaattgtagtaaagaagtaCCCCAACGCCGACTTCAGCTTT	15	AF261825 (Boyd et al. 2001)	This study
		SAL-70-D	1.3	P-TGAAGGTTCGACAGCACGTGC*TCCCTTTAGTGAGGGTTAAT*			
MLVA locus STTR10	PSLT064	SAL-74-U	1.1	*TAATACGACTCACTATAGGG*tatgaatgttattgtgtgttgattCGGGCGCGGCTGGAGTATTTG	48	AE006471 (McClelland et al. 2001)	(Lindstedt et al. 2004)
		SAL-74-D	1.1	P-CGCAACTCCCGGACAAGAAT*TCCCTTTAGTGAGGGTTAAT*			

Primer (T7 and T3), anti-TAG, target-specific sequences and SNP positions are indicated by, respectively, italic, lower-case, underlined and boldface sequences. P: phosphate

Table S3 Probes used in MOL-PCR_2

Target	Targeted sequence	Probe	Probe pre-mix	Sequence (5’→3’)	Bead region	Accession no.	Reference of target-specific sequence
Salmonella enterica subsp. *enterica* serovar Typhimurium	*rpoB*	rpoB-U	2.2	*TAATACGACTCACTATAGGG*gtaattgaattgaaagataagtgtTTTCTCAGCTGCACCGTAG**C**	18	AE006468 (McClelland et al. 2001)	(Hernández Guijarro et al. 2012)
		rpoB-D	2.2	P-CCTGGCGTCTTCTTTGACTCC*TCCCTTTAGTGAGGGTTAAT*			
ST64T/P22	g17	SAL-12-U	-	*TAATACGACTCACTATAGGG*gtaagattagaagttaatgaagaaGCGTTGCAGTAATCAGGTTTG	52	AY052766 (Mmolawa et al. 2003)	(Fang et al. 2012)
		SAL-12-D	-	P-ATGTTGTTGTCATACTGAAATGC*TCCCTTTAGTGAGGGTTAAT*			
ST64T/P22	*gtrA*/*gtrB*	SAL-14-U	2.1	*TAATACGACTCACTATAGGG*atttgttatgataaatgtgtagtgCTTTCTCGGCAATTAGCCTGGTATG	42	AY052766 (Mmolawa et al. 2003)	(Ross and Heuzenroeder 2005)
		SAL-14-D	2.1	P-CGGCTTTATCTATTCCAGAT*TCCCTTTAGTGAGGGTTAAT*			
ST64T/P22	*gtrC*	SAL-15-U	2.1	*TAATACGACTCACTATAGGG*agagtattagtagttattgtaagtGCATTAACACCTCTGACCACATC	57	AY052766 (Mmolawa et al. 2003)	(Ross and Heuzenroeder 2005)
		SAL-15-D	2.1	P-CAATTATTGTTAATAATGCGTGG*TCCCTTTAGTGAGGGTTAAT*			
ST64T/P22	*int*	SAL-18-U	-	*TAATACGACTCACTATAGGG*aatgaaatagtgttaaatgagtgtCGGCAATCATCACAAATGG	74	NC_002371 (Pedulla et al. 2003)	(Fang et al. 2012)
		SAL-18-D	-	P-GTGTTCGTCTACAAGGAAAGC*TCCCTTTAGTGAGGGTTAAT*			
P22	*sieB*	SAL-20-U	2.1	*TAATACGACTCACTATAGGG*tttgttagaatgagaagatttatgACAACTCATGGTGGCAGGAG	75	NC_002371 (Pedulla et al. 2003)	(Ross and Heuzenroeder 2005)
		SAL-20-D	2.1	P-CTAATGCGTTTTTTCCTGCA*TCCCTTTAGTGAGGGTTAAT*			
ST64T/P22	*eac*	SAL-21-U	2.1	*TAATACGACTCACTATAGGG*aaagaattagtatgatagatgagaCAGCTCTTTGTTGTATGCGC	76	NC_002371 (Pedulla et al. 2003)	(Fang et al. 2012)
		SAL-21-D	2.1	P-GGCCTTCCTTTGTGTTTCCC*TCCCTTTAGTGAGGGTTAAT*			
P22	g13	SAL-24-U	2.1	*TAATACGACTCACTATAGGG*tattagagtttgagaataagtagtCAGCGAAGCGTTTGATTAG	33	NC_002371 (Pedulla et al. 2003)	(Mikasová et al. 2005)
		SAL-24-D	2.1	P-CGAACCAATCGAGTCTGTG*TCCCTTTAGTGAGGGTTAAT*			
ST104	g44	SAL-26-U	2.1	*TAATACGACTCACTATAGGG*tttgatttaagagtgttgaatgtaCAACGCCCCACACACCA	26	AB102868 (Tanaka et al. 2004)	(Fang et al. 2012)
		SAL-26-D	2.1	P-GGTTCGGTACCACCTTTAATG*TCCCTTTAGTGAGGGTTAAT*			
ST104	g61	SAL-27-U	2.2	*TAATACGACTCACTATAGGG*aattgagaaagagataaatgatagCGCTACAGCAACAAAAACG	72	AB102868 (Tanaka et al. 2004)	(Drahovská et al. 2007)
		SAL-27-D	2.2	P-TATGCTCCAGATGGAAGAGAGG*TCCCTTTAGTGAGGGTTAAT*			

Table S3 (continued)

Target	Targeted sequence	Probe	Probe pre-mix	Sequence (5’→3’)	Bead region	Accession no.	Reference of target-specific sequence
AFLP fragments	CA-3	SAL-35-U	2.1	*TAATACGACTCACTATAGGG*aataagagaattgatatgaagatgAATGGCTGGCAGGGTCTGTTC	35	AF500155 (Hu et al. 2002)	(Fang et al. 2012)
		SAL-35-D	2.1	P-GAACCTGACGGACAGGCG*TCCCTTTAGTGAGGGTTAAT*			
AFLP fragments	CA-7	SAL-36-U	2.2	*TAATACGACTCACTATAGGG*attgtgaaagaaagagaagaaattCATCGGGGTGATCAGCA	14	AF500159 (Hu et al. 2002)	(Fang et al. 2012; Lan et al. 2007)
		SAL-36-D	2.2	P-TGATTACCCTGTTTGCCCG*TCCCTTTAGTGAGGGTTAAT*			
AFLP fragments	CA26.1	SAL-37-U	2.2	*TAATACGACTCACTATAGGg*tatagtgtgattagatttgtaaaGCGGCGCGTATTTCGTGC	78	DQ835569 (Lan et al. 2007)	(Lan et al. 2007)
		SAL-37-D	2.2	P-ATCGCATTGTCATTACCAGCA*TCCCTTTAGTGAGGGTTAAT*			
AFLP fragments	CA28.4	SAL-38-U	2.2	*TAATACGACTCACTATAGGG*attaagtaagaattgagagtttgaTGATGCACTAATTCGTCG	21	DQ835568 (Lan et al. 2007)	(Lan et al. 2007)
		SAL-38-D	2.2	P-CGAGGCTGCTGGATATATTC*TCCCTTTAGTGAGGGTTAAT*			
AFLP fragments	CG-1	SAL-39-U	2.2	*TAATACGACTCACTATAGGG*aaattagttgaaagtatgagaaagCAGGGAACCGTCTTGAG	20	AF500162 (Hu et al. 2002)	(Lan et al. 2007)
		SAL-39-D	2.2	P-CAAGTTCAGAGCGCAATGAC*TCCCTTTAGTGAGGGTTAAT*			
AFLP fragments	CG-2	SAL-40-U	-	*TAATACGACTCACTATAGGG*tattagagagaaattgtagagattCGCTATATCACGCTGAACAGAC	61	AF500163 (Hu et al. 2002)	(Lan et al. 2007)
		SAL-40-D	-	P-CAGAACTAGGTGAAACAAAAGAGG*TCCCTTTAGTGAGGGTTAAT*			
AFLP fragments	CT-1	SAL-42-U	-	*TAATACGACTCACTATAGGG*tattgttgaatgtgtttaaagagaCATCTGCTGATAGCTTAGTTGTC	47	AF500164 (Hu et al. 2002)	(Lan et al. 2007)
		SAL-42-D	-	P-GATAATGCCAACGACAATGCAGG*TCCCTTTAGTGAGGGTTAAT*			
AFLP fragments	CT-2	SAL-43-U	2.2	*TAATACGACTCACTATAGGGg*tatgttgtaatgtattaagaaagCCCAGGTAAACAGGAAATCCA	25	AF500165 (Hu et al. 2002)	(Fang et al. 2012)
		SAL-43-D	2.2	P-TTCCGGCACTGAAAATACTGC*TCCCTTTAGTGAGGGTTAAT*			
AFLP fragments	GA27.1	SAL-47-U	2.2	TAATACGACTCACTATAGGGaagatgatagttaagtgtaagttaGGTTTGTCCTACGACCCC	27	AY052766 (Mmolawa et al. 2003)	(Fang et al. 2012)
		SAL-47-D	2.2	P-GGAATCCTTCCATCGGAAATGA*TCCCTTTAGTGAGGGTTAAT*			
AFLP fragments	GC-1	SAL-48-U	2.2	*TAATACGACTCACTATAGGG*tgagtaagtttgtatgtttaagtaTGGAAGAACAAGCAAACAAGATTC	65	AF500167 (Hu et al. 2002)	(Fang et al. 2012)
		SAL-48-D	2.2	P-TCGTAGAACTACTGCAAAAAGCCAG*TCCCTTTAGTGAGGGTTAAT*			

Table S3 (continued)

Target	Targeted sequence	Probe	Probe pre-mix	Sequence (5’→3’)	Bead region	Accession no.	Reference of target-specific sequence
Streptomycin/ spectinomycin resistance	*aadA2*	SAL-66-U	-	*TAATACGACTCACTATAGGG*ttgtgtagttaagagttgtttaatGGTATCTTC GAGCCAGCCAT	36	AF261825 (Boyd et al. 2001)	This study
		SAL-66-D	-	P-GATCGACATTGATCTAGCTA*TCCCTTTAGTGAGGGTTAAT*			
Phase 2 flagellar gene	*fljB*	SAL-73-U	-	*TAATACGACTCACTATAGGG*tgaaatgtgtatttgtatgtttagCCAGCCGCA AGGGTTACTGTAC	62	AE006468 (McClelland et al. 2001)	(Muñoz et al. 2010)
		SAL-73-D	-	P-CGTCAGTAGCAACGTTAACTTCATAA*TCCCTTTAGTGAG GGTTAAT*			

Primer (T7 and T3), anti-TAG, target-specific sequences and SNP positions are indicated by, respectively, italic, lower-case, underlined and boldface sequences. P: phosphate

Table S4 Probes used in MOL-PCR_SNP

SNP	Targeted sequence	Probe	Probe pre-mix	Sequence (5’→3’)	Bead region	Accession no.	Reference of SNP
TM81	STM0080	SAL-56-U_SNP	S.1	*TAATACGACTCACTATAGGG*aattagaagtaagtagagtttaagGGTCGATAACGATACCGGTATG**A**	56	AE006468 (McClelland et al. 2001)	(Hu et al. 2006)
		SAL-56-U_WT	S.2	*TAATACGACTCACTATAGGG*tttgatttaagagtgttgaatgtaGGTCGATAACGATACCGGTATG**G**	26		
		SAL-56-D	S.1	P-TGTCTTACAAAAACGCCGCC*TCCCTTTAGTGAGGGTTAAT*			
TM3230_1	intergenic	SAL-58-U_SNP	-	*TAATACGACTCACTATAGGG*ttgtgatagtagttagatatttgtGGCTTTGACGAAGACTAAACC**C**	39	AE006468 (McClelland et al. 2001)	(Hu et al. 2006)
		SAL-58-U_WT	S.3	*TAATACGACTCACTATAGGg*tgtgttatttgtttgtaaagtatGGCTTTGACGAAGACTAAACC**T**	19		
		SAL-58-D	S.3	P-CCCCCTCTATACCCTATTTC*TCCCTTTAGTGAGGGTTAAT*			
TM1231	STM1269	SAL-59-U_SNP	S.1	*TAATACGACTCACTATAGGG*tttgttgttaagtatgtgatttagCCAGCGCTTGATGGTCGGC**T**	63	AE006468 (McClelland et al. 2001)	(Hu et al. 2006)
		SAL-59-U_WT	S.2	*TAATACGACTCACTATAGGG*tattagagtttgagaataagtagtCCAGCGCTTGATGGTCGGC**G**	33		
		SAL-59-D	S.1	P-CTTTCAGCCACGACGATATGGTG*TCCCTTTAGTGAGGGTTAAT*			
TM973	STM1002	SAL-60-U_SNP	S.1	*TAATACGACTCACTATAGGG*atgatgtgtttgatttgaattgaaGCTCATGTTCTGCTTATTAGCACCGAAGG**T**	64	AE006468 (McClelland et al. 2001)	(Hu et al. 2006)
		SAL-60-U_WT	S.2	*TAATACGACTCACTATAGGG*attgtgaaagaaagagaagaaattCTGCTTATTAGCACCGAAGG**C**	14		
		SAL-60-D	S.1	P-GATACGTCCCCAGATATTTATGAAGATATAG*TCCCTTTAGTGAGGGTTAAT*			
1880	*otsA*	SAL-61-U_SNP	S.1	*TAATACGACTCACTATAGGG*tttgtgtgttattgtaattgagatCACGATTGAGCGCCGC**A**	67	AE006468 (McClelland et al. 2001)	(Pang et al. 2012)
		SAL-61-U_WT	S.2	*TAATACGACTCACTATAGGG*tatgaatgttattgtgtgttgattCACGATTGAGCGCCGC**C**	48		
		SAL-61-D	S.1	P-GCCACGTCATCCCGATCG*TCCCTTTAGTGAGGGTTAAT*			
2199	*napF*	SAL-62-U_SNP	S.1	*TAATACGACTCACTATAGGG*agtgaatgtaagattatgtatttgCGGAAGAAAAAGCGATTC**A**	13	AE006468 (McClelland et al. 2001)	(Pang et al. 2012)
		SAL-62-U_WT	S.2	*TAATACGACTCACTATAGGG*agagtattagtagttattgtaagtGAAGAAAAAGCGATTC**G**	57		
		SAL-62-D	S.1	P-GTACAGGCCTGCGCACAG*TCCCTTTAGTGAGGGTTAAT*			

Table S4 (continued)

SNP	Targeted sequence	Probe	Probe pre-mix	Sequence (5’→3’)	Bead region	Accession no.	Reference of SNP
101	*araD*	SAL-63-U_SNP	S.1	*TAATACGACTCACTATAGGG*attaagtaagaattgagagtttgaGGCGCGAATGGGTGTGTAC**A**	21	AE006468 (McClelland et al. 2001)	(Pang et al. 2012)
		SAL-63-U_WT	S.2	*TAATACGACTCACTATAGGG*aattgagaaagagataaatgatagCGAATGGGTGTGTAC**G**	72		
		SAL-63-D	S.1	P-ATGCCGCCGATAGTCGG*TCCCTTTAGTGAGGGTTAAT*			
TM3124_1	intergenic	SAL-64-U_SNP	S.1	*TAATACGACTCACTATAGGG*gatagatttagaatgaattaagtgTCACAACTTCAAAATAAAACGTTATAAATTAATA**T**	28	AE006468 (McClelland et al. 2001)	(Hu et al. 2006)
		SAL-64-U_WT	S.2	*TAATACGACTCACTATAGGG*aatgaaatagtgttaaatgagtgtTCACAACTTCAAAATAAAACGTTATAAATTAATA**G**	74		
		SAL-64-D	S.1	P-ATTATATCAACAATCGCTTTTATCCTTGC*TCCCTTTAGTGAGGGTTAAT*			
TM3275_2	intergenic	SAL-65-U_SNP	S.1	*TAATACGACTCACTATAGGG*agtagaaagttgaaattgattatgCCTGCAAATACTCGTACGGGTCGCG**C**	12	AE006468 (McClelland et al. 2001)	(Hu et al. 2006)
		SAL-65-U_WT	S.2	*TAATACGACTCACTATAGGG*tttgttagaatgagaagatttatgCCTGCAAATACTCGTACGGGTCGCG**G**	75		
		SAL-65-D	S.1	P-TCTTTTACATCATTACGACGTCAAAC*TCCCTTTAGTGAGGGTTAAT*			
4213	*yjeF*	SAL-71-U_SNP	S.1	*TAATACGACTCACTATAGGg*tatgttgtaatgtattaagaaagTCTGGCGATTATTGGCGG**T**	25	AE006468 (McClelland et al. 2001)	(Pang et al. 2012)
		SAL-71-U_WT	S.2	*TAATACGACTCACTATAGGg*tttgtgtttgtataagttgttaaTCTGGCGATTATTGGCGG**C**	53		
		SAL-71-D	S.1	P-GACCAGGGAACAGCGGGCGC*TCCCTTTAGTGAGGGTTAAT*			
TM2079	intergenic	SAL-72-U_SNP	S.3	*TAATACGACTCACTATAGGG*aatgtaaagtaaagaaagtgatgaTACATCAGGCAACGGTACG**T**	44	AE006468 (McClelland et al. 2001)	(Hu et al. 2006)
		SAL-72-U_WT	S.3	*TAATACGACTCACTATAGGG*atttgttatgataaatgtgtagtgATACATCAGGCAACGGTACG**A**	42		
		SAL-72-D	S.3	P-CTATAGGACACCGCGCTAAG*TCCCTTTAGTGAGGGTTAAT*			

Primer (T7 and T3), anti-TAG, target-specific sequences and SNP positions are indicated by, respectively, italic, lower-case, underlined and boldface sequences. P: phosphate

Table S5 Specificity of the internal positive control probes

Genus	Species	Subspecies	Serovar	Reference[a]	*invA*	*rpoB*
Escherichia	*coli*	NA	NA	ATCC 25922	-	-
Listeria	*monocytogenes*	NA	NA	ATCC 51772	-	-
Shigella	*sonneï*	NA	NA	10-03865	-	-
Salmonella	*enterica*	*enterica*	Agona	NH.II.18.12	+	-
Salmonella	*enterica*	*enterica*	Derby	H.II.34.34	+	-
Salmonella	*enterica*	*enterica*	Enteritidis	H.VI.6.32	+	-
Salmonella	*enterica*	*enterica*	Infantis	NH.III.66.30	+	-
Salmonella	*enterica*	*enterica*	Kentucky	H.I.70.26	+	-
Salmonella	*enterica*	*enterica*	Newport	H.V.70.42	+	-
Salmonella	*enterica*	*enterica*	Paratyphi B	H.VII.64.65	+	-
Salmonella	*enterica*	*enterica*	Typhi	H.III.28.24	+	-
Salmonella	*enterica*	*enterica*	Typhimurium	H.II.32.32	+	+
Salmonella	*enterica*	*enterica*	Typhimurium var. Copenhagen	H.II.13.13	+	+

+ positive reaction in MOL-PCR; - negative reaction in MOL-PCR

[a] Purified DNA was available in our laboratory (Barbau-Piednoir et al. 2013)

Table S6 Minimum and maximum of observed signal-to-noise ratios, calculated cut-off values and assigned prime numbers for the markers in MOL-PCR_1

Marker	Negative samples		Positive samples		Calculated cut-off value	Assigned prime number
	Minimum SN	Maximum SN	Minimum SN	Maximum SN		
invA	NA	NA	8.15	27.66	8	2
rpoB	NA	NA	10.45	47.59	10	2
SAL-10	0.64	2.83	3.82	30.31	3.5	7
SAL-11	0.60	2.54	7.66	28.62	5	13
SAL-16	0.39	2.51	4.05	28.12	3.5	17
SAL-23	0.64	2.66	8.90	33.98	6	11
SAL-25	0.76	2.93	8.82	29.40	6	31
SAL-29	0.72	2.77	3.87	31.78	3.5	5
SAL-33	0.42	2.19	4.67	18.69	3.5	67
SAL-45	0.51	2.64	5.13	28.60	4	19
SAL-49	0.72	2.79	6.93	30.38	5	3
SAL-50	0.63	2.78	6.17	24.37	4	43
SAL-51	0.72	2.67	5.82	20.00	4	47
SAL-53	0.23	2.49	6.63	31.33	5	37
SAL-55	0.72	2.81	4.31	21.29	3.5	41
SAL-67	0.57	2.33	10.01	39.68	6	53
SAL-68	0.34	2.39	4.54	18.55	3.5	61
SAL-69	0.37	2.25	7.30	21.88	5	59
SAL-70	0.51	2.45	12.37	33.85	7	29
SAL-74	0.67	2.55	10.15	31.22	6	23

SN: signal-to-noise ratio; NA: not applicable

Table S7 Minimum and maximum of observed signal-to-noise ratios, calculated cut-off values and assigned prime numbers for the markers in MOL-PCR_2

Marker	Negative samples		Positive samples		Calculated cut-off value	Assigned prime number
	Minimum SN	Maximum SN	Minimum SN	Maximum SN		
rpoB	NA	NA	4.85	22.02	4	2
SAL-12	0.70	2.26	5.34	15.10	4	41
SAL-14	0.67	2.23	4.10	17.46	3	23
SAL-15	0.55	1.91	3.35	22.65	3	7
SAL-18	0.70	2.33	3.20	17.03	3	11
SAL-20	0.52	2.01	4.48	16.80	3	17
SAL-21	0.54	2.10	3.93	15.77	3	53
SAL-24	0.29	1.78	3.27	13.22	3	59
SAL-26	0.70	2.45	3.62	14.82	3	29
SAL-27	0.69	2.21	3.13	13.77	3	13
SAL-35	0.55	1.99	3.14	12.18	3	19
SAL-36	0.60	1.96	13.23	26.13	8	71
SAL-37	0.48	2.94	5.28	10.58	4	61
SAL-38	0.61	2.15	4.72	12.31	3.5	67
SAL-39	0.61	1.96	7.11	22.12	5	37
SAL-40	0.41	1.96	3.14	11.55	3	73
SAL-42	0.55	2.23	3.02	9.88	3	5
SAL-43	0.43	2.16	7.36	15.93	5	79
SAL-47	0.36	1.65	3.21	16.40	3	47
SAL-48	0.49	1.81	5.05	18.16	3.5	31
SAL-66	0.33	2.44	7.79	28.81	5	43
SAL-73	0.77	2.05	10.88	25.10	6	3

SN: signal-to-noise ratio; NA: not applicable

Table S8 Minimum and maximum of median fluorescence intensity, calculated cut-off values and assigned prime numbers for the markers in MOL-PCR_SNP

Marker	Negative samples		Positive samples		Calculated cut-off value	Assigned prime number – presence of SNP	Assigned prime number – absence of locus
	Minimum MFI	Maximum MFI	Minimum MFI	Maximum MFI			
SAL-56_SNP	106	357	771	2063	600	5	47
SAL-56_WT	189	485	737.5	2342	600	1	
SAL-58_SNP	103	422.5	624.5	1423	500	29	41
SAL-58_WT	290	590	885	2319.5	700	1	
SAL-59_SNP	83	311.5	407	1607	350	11	53
SAL-59_WT	93	240.5	440	2154.5	350	1	
SAL-60_SNP	179	769.5	1243	2407.5	1000	3	59
SAL-60_WT	108	259	463	1556	350	1	
SAL-61_SNP	71	230.5	251.5	726.5	250	13	61
SAL-61_WT	163	558	1106	2994	800	1	
SAL-62_SNP	71	302	385	1106	350	19	67
SAL-62_WT	99.5	182.5	217.5	1110	200	1	
SAL-63_SNP	90	500	985	2091	700	17	71
SAL-63_WT	118	360	409	1955.5	400	1	
SAL-64_SNP	99	540.5	803	1193	700	31	73
SAL-64_WT	118	168	217	1543	200	1	
SAL-65_SNP	102	383	1153	2072	800	7	79
SAL-65_WT	97	242	600	1699	400	1	
SAL-71_SNP	204	810.5	1565	2411	1200	23	43
SAL-71_WT	117	227	431	1558	350	1	
SAL-72_SNP	114	443	1908	2027.5	1200	37	83
SAL-72_WT	158	165	319	1914	250	1	

MFI: median fluorescence intensity

Table S9 In-house code for MOL-PCR_1

Unique GPP	In-house code
3	1
15	2
69	3
87	4
123	5
345	6
435	7
795	8
851	9
10005	10
15015	11
19095	12
23055	13
34891	14
248235	15
255255	16
345345	17
1312311	18
5870865	19
6561555	20
19114095	21
23138115	22
43444401	23
150345195	24
324939615	25
574893165	26
3457939485	27
1.0028E+11	28
2.04963E+16	29
1.20928E+18	30
9.54E+19	31
1.06E+20	32
3.91E+21	33

Table S10 In-house code for MOL-PCR_2

Unique GPP	In-house code
1	1
3	2
43	3
57	4
129	5
82203	6
97055	7
291165	8
465465	9
680295	10
1228371	11
8843835	12
17606651	13
23339049	14
224989611	15
430422405	16
8178025695	17
10874497865	18
32623493595	19
66371935245	20
1.54007E+11	21
1.69216E+11	22
2.58277E+11	23
9.23053E+11	24
1.40281E+12	25
3.62117E+12	26
4.90726E+12	27
1.11059E+13	28
1.76239E+14	29
4.50435E+17	30

Table S11 In-house code for MOL-PCR_SNP

Unique GPP	In-house code
1	1
29	2
37	3
41	4
69	5
105	6
221	7
1155	8
2139	9
4199	10
180557	11

Data set S1 *Salmonella enterica* subsp. *enterica* serovar Typhimurium and 1,4,[5],12:i:- isolates used in the development and validation of the multiplex oligonucleotide ligation-PCR (MOL-PCR) and their subtyping data.

Sample_ID	Serovar	Source	Outbreak_related	PCR_screening	Stability_experiment	PFGE_cluster[a]	GPP_MOL-PCR_1	GPP_MOL-PCR_2	GPP_MOL-PCR_SNP	MOL-PCR_code	Phage_type[b]	MLVA
S0001	Typhimurium	Human	0	1	1	NT	3.91E+21	1.11059E+13	4199	33-28-10	DT104	3-15-10-23-311
S0002	Typhimurium	Human	0	1	1	NT	248235	224989611	1	15-15-1	DT120	3-13-10-NA-211
S0003	Typhimurium	Human	0	1	0	NT	19114095	32623493595	1	21-19-1	DT120	3-13-10-NA-211
S0004	Typhimurium	Human	0	1	0	NT	15	3	1	2-2-1	DT120	3-12-10-NA-211
S0005	Typhimurium	Human	0	1	0	B3	255255	8843835	1155	16-12-8	DT35	3-15-11-NA-311
S0006	Typhimurium	Human	0	1	1	NT	3.91E+21	1.11059E+13	4199	33-28-10	DT104	3-15-10-23-311
S0007	Typhimurium	Human	0	1	1	NT	345345	8178025695	2139	17-17-9	U302	4-14-18-7-211
S0008	1,4,[5],12:i:-	Human	0	1	0	NT	15	1	1	2-1-1	DT193	3-13-10-NA-211
S0009	Typhimurium	Human	0	1	1	NT	15	3	1	2-2-1	DT120	3-12-11-NA-211
S0010	Typhimurium	Human	0	1	0	NT	3.91E+21	1.11059E+13	4199	33-28-10	DT12	3-17-16-11-NA
S0011	1,4,[5],12:i:-	Human	0	1	1	NT	15	1	1	2-1-1	DT193	3-13-9-NA-211
S0012	Typhimurium	Human	0	1	0	NT	3.91E+21	1.11059E+13	4199	33-28-10	DT104	3-13-12-22-311
S0013	Typhimurium	Human	0	1	0	NT	15	3	1	2-2-1	NT	3-12-11-NA-211
S0014	1,4,[5],12:i:-	Human	0	1	1	NT	15	1	1	2-1-1	DT120	3-13-10-NA-211
S0015	Typhimurium	Human	0	1	0	A3	15	3	1	2-2-1	DT193	3-12-11-NA-211
S0016	Typhimurium	Human	0	1	1	NT	255255	8843835	1155	16-12-8	DT195	3-15-13-NA-311
S0017	Typhimurium	Human	0	1	1	NT	255255	8843835	1155	16-12-8	U302	3-16-10-NA-311
S0018	1,4,[5],12:i:-	Human	0	1	0	NT	15	1	1	2-1-1	RDNC	3-12-11-NA-211
S0019	Typhimurium	Human	0	1	1	NT	255255	8843835	1155	16-12-8	DT12	3-14-10-NA-311
S0020	1,4,[5],12:i:-	Human	0	1	1	NT	15	1	1	2-1-1	DT195	3-13-10-NA-211
S0021	Typhimurium	Human	0	1	0	NT	255255	8843835	1155	16-12-8	DT195	3-16-10-NA-311
S0022	1,4,[5],12:i:-	Human	0	1	1	A10	15	1	1	2-1-1	DT195	3-13-10-NA-211
S0023	Typhimurium	Human	0	1	0	NT	345345	430422405	69	17-16-5	U302	4-8-10-9-211
S0024	Typhimurium	Human	0	1	1	B1	255255	8843835	1155	16-12-8	U302	3-15-11-NA-311
S0025	Typhimurium	Human	0	1	1	NT	345345	8178025695	2139	17-17-9	DT12	5-14-11-8-211
S0026	1,4,[5],12:i:-	Human	0	1	0	NT	15	1	1	2-1-1	DT193	3-12-10-NA-211
S0027	Typhimurium	Human	0	1	1	NT	3.91E+21	1.11059E+13	4199	33-28-10	DT104	3-14-11-21-311
S0028	Typhimurium	Human	0	1	1	NT	255255	8843835	1155	16-12-8	DT193	3-15-13-NA-311
S0029	Typhimurium	Human	0	1	1	NT	255255	8843835	1155	16-12-8	DT193	3-15-13-NA-311
S0030	Typhimurium	Human	0	1	1	NT	345345	8178025695	2139	17-17-9	DT12	5-13-15-8-211
S0031	Typhimurium	Human	0	0	0	NT	851	3	221	9-2-7	DT1	3-14-10-15-NA
S0032	Typhimurium	Human	0	0	0	NT	851	3	221	9-2-7	DT1	3-14-10-15-311
S0033	Typhimurium	Human	0	0	0	NT	3.91E+21	1.11059E+13	4199	33-28-10	U302	3-14-18-14-311
S0034	Typhimurium	Human	0	0	0	NT	255255	8843835	1155	16-12-8	DT35	3-14-10-NA-311
S0035	Typhimurium	Human	0	0	1	NT	3.91E+21	1.11059E+13	4199	33-28-10	DT12	3-14-18-14-311
S0036	Typhimurium	Human	0	0	0	NT	3	1228371	105	1-11-6	DT1	3-19-5-NA-311
S0037	Typhimurium	Human	0	0	1	NT	3.91E+21	1.11059E+13	4199	33-28-10	U302	3-18-16-17-311
S0038	Typhimurium	Human	0	0	1	NT	255255	8843835	1155	16-12-8	DT195	3-14-10-NA-311
S0039	Typhimurium	Human	0	0	1	NT	15	3	1	2-2-1	DT120	3-12-9-NA-211
S0040	Typhimurium	Human	0	0	1	A3	15	3	1	2-2-1	DT195	3-12-11-NA-211

Sample_ID	Serovar	Source	Outbreak_related	PCR_screening	Stability_experiment	PFGE_cluster[a]	GPP_MOL-PCR_1	GPP_MOL-PCR_2	GPP_MOL-PCR_SNP	MOL-PCR_code	Phage_type[b]	MLVA
S0041	Typhimurium	Human	0	0	0	NT	34891	3	4199	14-2-10	DT1	3-11-16-23-311
S0042	Typhimurium	Human	0	0	0	NT	34891	3	4199	14-2-10	DT1	3-11-16-23-311
S0043	Typhimurium	Human	0	0	0	NT	34891	3	4199	14-2-10	DT1	3-11-16-23-311
S0044	Typhimurium	Human	0	0	0	NT	255255	3.62117E+12	1155	16-26-8	DT208	3-15-13-NA-311
S0045	1,4,[5],12:i:-	Human	0	0	0	A5	15	1	1	2-1-1	U311	3-15-13-NA-311
S0046	Typhimurium	Human	0	0	0	NT	255255	8843835	1155	16-12-8	DT193	3-15-13-NA-311
S0047	Typhimurium	Human	0	0	0	NT	255255	3.62117E+12	1155	16-26-8	DT195	3-15-13-NA-311
S0048	Typhimurium	Human	0	0	0	NT	255255	8843835	1155	16-12-8	DT195	3-15-13-NA-311
S0049	Typhimurium	Human	0	0	0	NT	34891	3	4199	14-2-10	DT1	3-11-16-23-NA
S0050	Typhimurium	Human	0	0	0	NT	34891	1228371	4199	14-11-10	DT1	3-11-15-22-NA
S0051	Typhimurium	Human	0	0	0	NT	255255	8843835	1155	16-12-8	DT193	3-15-12-NA-311
S0052	1,4,[5],12:i:-	Human	0	0	0	A10	15	1	1	2-1-1	DT193	3-12-9-NA-211
S0053	Typhimurium	Human	0	0	0	NT	3.91E+21	1.11059E+13	4199	33-28-10	DT104	3-14-13-20-311
S0054	1,4,[5],12:i:-	Human	0	0	0	NT	15	1	1	2-1-1	DT110	3-12-8-NA-211
S0055	1,4,[5],12:i:-	Human	0	0	0	NT	15	1	1	2-1-1	DT110	3-11-12-NA-211
S0056	Typhimurium	Human	0	0	0	B2	255255	8843835	1155	16-12-8	DT208	3-10-7-NA-311
S0057	Typhimurium	Human	0	0	0	NT	19114095	1.40281E+12	1	21-25-1	DT193	3-15-9-NA-211
S0058	1,4,[5],12:i:-	Human	0	0	0	NT	15	1	1	2-1-1	RDNC	3-12-12-NA-211
S0059	1,4,[5],12:i:-	Human	0	0	0	A1	15	1	1	2-1-1	RDNC	3-13-11-NA-211
S0060	Typhimurium	Human	0	0	0	NT	324939615	9.23053E+11	1	25-24-1	DT177	3-17-8-NA-211
S0061	1,4,[5],12:i:-	Human	0	0	0	NT	19114095	10874497865	1	21-18-1	DT120	3-12-9-NA-211
S0062	1,4,[5],12:i:-	Human	0	0	0	NT	15	1	1	2-1-1	DT193	3-14-11-NA-211
S0063	Typhimurium	Human	0	0	0	NT	15	3	1	2-2-1	RDNC	3-14-6-NA-211
S0064	Typhimurium	Human	0	0	0	NT	15	3	1	2-2-1	DT193	3-12-10-NA-211
S0065	Typhimurium	Human	0	0	0	NT	255255	8843835	1155	16-12-8	DT193	3-15-13-NA-311
S0066	1,4,[5],12:i:-	Human	0	0	0	NT	15	1	1	2-1-1	DT193	3-14-11-NA-211
S0067	Typhimurium	Human	0	0	0	NT	255255	8843835	1155	16-12-8	DT193	3-16-12-NA-311
S0068	1,4,[5],12:i:-	Human	0	0	0	NT	15	1	1	2-1-1	RDNC	3-13-11-NA-211
S0069	1,4,[5],12:i:-	Human	0	0	0	NT	15	1	1	2-1-1	RDNC	3-12-12-NA-211
S0070	Typhimurium	Human	0	0	0	NT	15	3	1	2-2-1	DT193	3-12-10-NA-211
S0071	1,4,[5],12:i:-	Human	0	0	0	NT	15	1	1	2-1-1	DT193	3-13-9-NA-211
S0072	1,4,[5],12:i:-	Human	0	0	0	NT	15	1	1	2-1-1	DT193	3-11-10-NA-211
S0073	1,4,[5],12:i:-	Human	0	0	0	NT	15	1	1	2-1-1	RDNC	3-12-8-NA-211
S0074	Typhimurium	Human	0	0	0	NT	255255	8843835	1155	16-12-8	DT193	3-16-12-NA-311
S0075	Typhimurium	Human	0	0	0	NT	19114095	1.40281E+12	1	21-25-1	DT193	3-13-8-NA-211
S0076	Typhimurium	Human	0	0	0	B3	255255	8843835	1155	16-12-8	DT193	3-15-11-NA-311
S0077	Typhimurium	Human	0	0	0	NT	255255	8843835	1155	16-12-8	DT193	3-16-10-NA-311
S0078	Typhimurium	Human	0	0	0	NT	255255	8843835	1155	16-12-8	DT193	3-15-12-NA-311
S0079	Typhimurium	Human	0	0	0	NT	255255	8843835	1155	16-12-8	DT193	3-17-10-NA-211
S0080	Typhimurium	Human	0	0	0	A7	15	3	1	2-2-1	U311	3-12-10-NA-211
S0081	1,4,[5],12:i:-	Human	0	0	0	NT	15	1	1	2-1-1	DT138	3-12-9-NA-211
S0082	1,4,[5],12:i:-	Human	0	0	0	NT	15	1	1	2-1-1	RDNC	3-12-12-NA-211
S0083	1,4,[5],12:i:-	Human	0	0	0	NT	15	1	1	2-1-1	RDNC	3-12-9-NA-211

Sample_ID	Serovar	Source	Outbreak_related	PCR_screening	Stability_experiment	PFGE_cluster[a]	GPP_MOL-PCR_1	GPP_MOL-PCR_2	GPP_MOL-PCR_SNP	MOL-PCR_code	Phage_type[b]	MLVA
S0084	1,4,[5],12:i:-	Human	0	0	0	NT	15	1	1	2-1-1	DT193	3-13-8-NA-211
S0085	Typhimurium	Human	0	0	0	NT	3.91E+21	1.11059E+13	4199	33-28-10	DT104	3-15-14-17-311
S0086	Typhimurium	Human	0	0	0	NT	345	57	29	6-4-2	DT2	2-11-7-16-312
S0087	1,4,[5],12:i:-	Human	0	0	0	NT	15	1	1	2-1-1	DT193	3-8-9-NA-211
S0088	Typhimurium	Human	0	0	0	A11	15	3	1	2-2-1	DT110	3-12-11-NA-211
S0089	Typhimurium	Human	0	0	0	NT	255255	8843835	1155	16-12-8	NT	3-15-10-NA-311
S0090	Typhimurium	Human	0	0	0	B3	255255	8843835	1155	16-12-8	U302	3-17-11-NA-211
S0091	Typhimurium	Human	0	0	0	NT	15	3	1	2-2-1	DT120	3-12-10-NA-211
S0092	Typhimurium	Human	0	0	0	NT	19114095	66371935245	1	21-20-1	DT193	3-15-11-NA-211
S0093	1,4,[5],12:i:-	Human	0	0	0	NT	15	1	1	2-1-1	DT193	3-14-10-NA-211
S0094	1,4,[5],12:i:-	Human	0	0	0	NT	15	1	1	2-1-1	DT193	3-8-9-NA-211
S0095	Typhimurium	Human	0	0	0	NT	15015	680295	1155	11-10-8	DT12	3-14-7-NA-311
S0096	1,4,[5],12:i:-	Human	0	0	0	NT	15	1	1	2-1-1	DT193	3-12-9-NA-211
S0097	Typhimurium	Human	0	0	0	NT	255255	8843835	1155	16-12-8	DT193	3-14-11-NA-311
S0098	Typhimurium	Human	0	0	0	B3	255255	8843835	1155	16-12-8	DT193	3-17-11-NA-211
S0099	Typhimurium	Human	0	0	0	NT	15	3	1	2-2-1	DT110	3-17-10-NA-211
S0100	Typhimurium	Human	0	0	0	NT	43444401	465465	37	23-9-3	DT120	5-10-12-8-211
S0101	Typhimurium	Human	0	0	0	NT	255255	8843835	1155	16-12-8	DT193	3-15-10-NA-311
S0102	Typhimurium	Human	0	0	0	NT	15	3	1	2-2-1	RDNC	3-13-10-NA-211
S0103	1,4,[5],12:i:-	Human	0	0	0	NT	15	1	1	2-1-1	NT	3-13-9-NA-211
S0104	1,4,[5],12:i:-	Human	0	0	0	A13	15	1	1	2-1-1	DT138	3-13-10-NA-211
S0105	1,4,[5],12:i:-	Human	0	0	0	NT	15	1	1	2-1-1	DT193	3-12-8-NA-211
S0106	Typhimurium	Human	0	0	0	NT	255255	8843835	1155	16-12-8	DT193	3-15-10-NA-311
S0107	1,4,[5],12:i:-	Human	0	0	0	NT	15	1	1	2-1-1	DT120	3-12-10-NA-211
S0108	Typhimurium	Human	0	0	0	NT	255255	8843835	1155	16-12-8	DT193	3-15-10-NA-311
S0109	Typhimurium	Human	0	0	0	NT	5870865	8843835	69	19-12-5	DT194	3-11-6-8-211
S0110	Typhimurium	Human	0	0	0	A9	15	3	1	2-2-1	RDNC	3-12-11-NA-211
S0111	Typhimurium	Human	0	0	0	NT	255255	8843835	1155	16-12-8	DT193	3-15-10-NA-311
S0112	Typhimurium	Human	0	0	0	NT	69	3	29	3-2-2	DT8	2-9-NA-12-212
S0113	1,4,[5],12:i:-	Human	0	0	0	A8	15	1	1	2-1-1	DT110	3-12-9-NA-211
S0114	Typhimurium	Human	0	0	0	NT	3.91E+21	1.11059E+13	4199	33-28-10	DT104	3-14-12-22-311
S0115	Typhimurium	Human	0	0	1	NT	15	3	1	2-2-1	DT120	3-13-9-NA-211
S0116	Typhimurium	Human	0	0	0	NT	15	3	1	2-2-1	DT120	3-11-12-NA-211
S0117	Typhimurium	Human	0	0	0	B3	255255	8843835	1155	16-12-8	DT194	3-17-11-NA-211
S0118	Typhimurium	Human	0	0	0	NT	255255	8843835	1155	16-12-8	DT193	3-17-11-NA-211
S0119	1,4,[5],12:i:-	Human	0	0	0	A2	15	1	1	2-1-1	DT120	3-13-10-NA-211
S0120	Typhimurium	Human	0	0	0	NT	15	3	1	2-2-1	DT120	3-13-11-NA-211
S0121	Typhimurium	Human	0	0	0	NT	15	3	1	2-2-1	DT193	3-12-9-NA-211
S0122	1,4,[5],12:i:-	Human	0	0	0	NT	15	17606651	1	2-13-1	DT120	3-12-14-NA-211
S0123	Typhimurium	Human	0	0	0	NT	15	3	1	2-2-1	DT120	3-12-12-NA-211
S0124	Typhimurium	Human	0	0	0	NT	255255	8843835	1155	16-12-8	DT193	3-15-12-NA-311
S0125	Typhimurium	Human	0	0	0	B3	255255	8843835	1155	16-12-8	NT	3-17-11-NA-211
S0126	Typhimurium	Human	0	0	0	NT	255255	8843835	1155	16-12-8	DT193	3-15-12-NA-311

Sample_ID	Serovar	Source	Outbreak_related	PCR_screening	Stability_experiment	PFGE_cluster[a]	GPP_MOL-PCR_1	GPP_MOL-PCR_2	GPP_MOL-PCR_SNP	MOL-PCR_code	Phage_type[b]	MLVA
S0127	Typhimurium	Human	0	0	0	NT	15	3	1	2-2-1	DT120	3-11-13-NA-211
S0128	Typhimurium	Human	0	0	0	NT	3.91E+21	1.11059E+13	4199	33-28-10	DT104	3-14-5-10-311
S0129	Typhimurium	Human	0	0	0	NT	345345	8178025695	29	17-17-2	RDNC	2-12-8-9-212
S0130	Typhimurium	Human	0	0	0	NT	19114095	32623493595	1	21-19-1	DT120	3-13-8-NA-211
S0131	Typhimurium	Human	0	0	0	NT	3.91E+21	1.11059E+13	4199	33-28-10	DT104	3-10-15-14-311
S0132	Typhimurium	Human	0	0	0	NT	19114095	32623493595	1	21-19-1	DT120	3-13-9-NA-211
S0133	Typhimurium	Human	0	0	0	NT	19114095	32623493595	1	21-19-1	DT120	3-14-9-NA-211
S0134	Typhimurium	Human	0	0	0	NT	123	3	1	5-2-1	RDNC	3-15-NA-NA-211
S0135	Typhimurium	Human	0	0	0	NT	255255	8843835	1155	16-12-8	DT195	3-15-12-NA-311
S0136	Typhimurium	Human	0	0	0	NT	15	3	1	2-2-1	DT120	3-12-9-NA-211
S0137	Typhimurium	Human	0	0	0	NT	255255	8843835	1155	16-12-8	DT195	3-14-11-NA-311
S0138	1,4,[5],12:i:-	Human	0	0	0	NT	15	1	1	2-1-1	RDNC	3-12-11-NA-211
S0139	Typhimurium	Human	0	0	0	NT	19114095	32623493595	1	21-19-1	DT120	3-14-9-NA-211
S0140	1,4,[5],12:i:-	Human	0	0	0	A4	15	1	1	2-1-1	DT120	NA-12-10-NA-211
S0141	Typhimurium	Human	0	0	0	NT	19114095	32623493595	1	21-19-1	DT116	3-13-8-NA-211
S0142	Typhimurium	Human	0	0	0	NT	19114095	1.40281E+12	1	21-25-1	DT116	3-14-9-NA-211
S0143	1,4,[5],12:i:-	Human	0	0	0	NT	15	1	1	2-1-1	DT195	3-9-10-NA-211
S0144	Typhimurium	Human	0	0	0	NT	255255	8843835	1155	16-12-8	DT195	3-16-10-NA-311
S0145	Typhimurium	Human	0	0	0	NT	255255	8843835	1155	16-12-8	DT195	3-15-9-NA-311
S0146	Typhimurium	Human	0	0	0	NT	15	3	1	2-2-1	DT120	3-12-12-NA-211
S0147	Typhimurium	Human	0	0	0	NT	19114095	1.40281E+12	1	21-25-1	DT195	3-14-7-NA-211
S0148	1,4,[5],12:i:-	Human	0	0	0	NT	15	1	1	2-1-1	DT193	3-8-9-NA-211
S0149	1,4,[5],12:i:-	Human	0	0	0	NT	15	1	1	2-1-1	DT195	3-12-8-NA-211
S0150	Typhimurium	Human	0	0	0	NT	19114095	32623493595	1	21-19-1	DT195	3-14-7-NA-211
S0151	1,4,[5],12:i:-	Human	0	0	0	NT	15	1	1	2-1-1	DT193	3-8-9-NA-211
S0152	Typhimurium	Human	0	0	0	NT	345	57	29	6-4-2	DT2	2-10-12-11-212
S0153	1,4,[5],12:i:-	Human	0	0	0	NT	15	1	1	2-1-1	DT193	3-8-9-NA-211
S0154	Typhimurium	Human	0	0	0	NT	87	3	105	4-2-6	DT193	3-16-NA-NA-311
S0155	1,4,[5],12:i:-	Human	0	0	0	NT	15	1	1	2-1-1	DT120	3-8-9-NA-211
S0156	Typhimurium	Human	0	0	0	NT	19114095	1.40281E+12	1	21-25-1	DT195	3-13-7-NA-211
S0157	1,4,[5],12:i:-	Human	0	0	0	NT	15	1	1	2-1-1	DT193	3-13-11-NA-211
S0158	1,4,[5],12:i:-	Human	0	0	0	NT	6561555	97055	29	20-7-2	DT193	2-11-5-7-212
S0159	Typhimurium	Human	0	0	0	NT	255255	8843835	1155	16-12-8	DT195	3-14-11-NA-311
S0160	1,4,[5],12:i:-	Human	0	0	0	NT	15	1	1	2-1-1	DT110	3-12-10-NA-211
S0161	Typhimurium	Human	0	0	1	B3	255255	8843835	1155	16-12-8	DT195	3-15-11-NA-311
S0162	Typhimurium	Human	0	0	0	NT	3.91E+21	1.11059E+13	4199	33-28-10	DT110	3-12-21-14-NA
S0163	1,4,[5],12:i:-	Human	0	0	0	NT	15	1	1	2-1-1	NT	3-11-9-NA-211
S0164	1,4,[5],12:i:-	Human	0	0	0	NT	15	1	1	2-1-1	DT193	3-12-9-NA-211
S0165	Typhimurium	Human	0	0	0	NT	3.91E+21	1.11059E+13	4199	33-28-10	DT104	3-12-21-14-NA
S0166	Typhimurium	Human	0	0	0	NT	15	3	1	2-2-1	DT120	3-12-11-NA-211
S0167	Typhimurium	Human	0	0	0	NT	3	3	105	1-2-6	DT10	3-16-NA-NA-311
S0168	Typhimurium	Human	0	0	1	NT	19114095	32623493595	1	21-19-1	DT120	3-13-9-NA-211
S0169	1,4,[5],12:i:-	Human	0	0	0	A10	15	1	1	2-1-1	DT193	3-12-9-NA-11

Sample_ID	Serovar	Source	Outbreak_related	PCR_screening	Stability_experiment	PFGE_cluster[a]	GPP_MOL-PCR_1	GPP_MOL-PCR_2	GPP_MOL-PCR_SNP	MOL-PCR_code	Phage_type[b]	MLVA
S0170	1,4,[5],12:i:-	Human	0	0	0	NT	15	1	1	2-1-1	DT120	3-11-9-NA-211
S0171	Typhimurium	Human	0	0	0	NT	3.91E+21	1.11059E+13	4199	33-28-10	DT104	3-12-21-14-NA
S0172	1,4,[5],12:i:-	Human	0	0	0	A10	15	1	1	2-1-1	DT193	3-12-9-NA-NA
S0173	Typhimurium	Human	0	0	0	NT	15	3	1	2-2-1	DT120	3-12-11-NA-211
S0174	1,4,[5],12:i:-	Human	0	0	0	NT	15	1	1	2-1-1	DT193	3-12-9-NA-211
S0175	1,4,[5],12:i:-	Human	0	0	0	NT	15	1	1	2-1-1	DT193	3-13-9-NA-211
S0176	Typhimurium	Human	0	0	0	NT	3.91E+21	1.11059E+13	4199	33-28-10	DT104	3-12-22-14-311
S0177	Typhimurium	Human	0	0	0	NT	345345	8178025695	29	17-17-2	RDNC	2-12-7-9-212
S0178	Typhimurium	Human	0	0	0	NT	255255	8843835	1155	16-12-8	DT193	3-16-10-NA-311
S0179	Typhimurium	Human	0	0	0	NT	3.91E+21	1.11059E+13	4199	33-28-10	DT104	3-12-21-14-NA
S0180	Typhimurium	Human	0	0	0	NT	3.91E+21	1.11059E+13	4199	33-28-10	DT104	3-15-12-14-311
S0181	Typhimurium	Human	0	0	0	NT	1.20928E+18	2.58277E+11	4199	30-23-10	DT104	3-16-16-13-311
S0182	Typhimurium	Human	0	0	0	NT	15	3	1	2-2-1	DT193	3-12-11-NA-211
S0183	Typhimurium	Human	0	0	0	NT	324939615	9.23053E+11	1	25-24-1	RDNC	3-16-9-NA-211
S0184	Typhimurium	Human	0	0	1	NT	255255	8843835	1155	16-12-8	U302	3-16-10-NA-311
S0185	1,4,[5],12:i:-	Human	0	0	0	NT	15	1	1	2-1-1	RDNC	3-12-15-NA-211
S0186	Typhimurium	Human	0	0	0	NT	3.91E+21	1.11059E+13	4199	33-28-10	DT104	3-12-22-14-311
S0187	1,4,[5],12:i:-	Human	0	0	0	NT	15	1	1	2-1-1	DT120	3-12-10-NA-211
S0188	Typhimurium	Human	0	0	0	NT	3.91E+21	1.11059E+13	4199	33-28-10	DT104	3-12-21-14-NA
S0189	Typhimurium	Human	0	0	0	NT	19114095	1.40281E+12	1	21-25-1	DT116	3-13-8-NA-211
S0190	Typhimurium	Human	0	0	0	NT	324939615	9.23053E+11	1	25-24-1	RDNC	3-16-9-NA-211
S0191	Typhimurium	Human	0	0	0	NT	3.91E+21	1.11059E+13	4199	33-28-10	DT104	3-12-21-14-311
S0192	1,4,[5],12:i:-	Human	0	0	0	NT	15	1	1	2-1-1	DT193	3-12-9-NA-211
S0193	Typhimurium	Human	0	0	0	NT	9.54E+19	1.11059E+13	4199	31-28-10	DT104	3-13-11-25-211
S0194	Typhimurium	Human	0	0	0	NT	3.91E+21	1.11059E+13	4199	33-28-10	DT104	3-12-22-14-311
S0195	1,4,[5],12:i:-	Human	0	0	0	A11	15	1	1	2-1-1	DT120	3-11-12-NA-NA
S0196	Typhimurium	Human	0	0	0	NT	19114095	1.40281E+12	1	21-25-1	DT116	3-13-8-NA-NA
S0197	Typhimurium	Human	0	0	0	NT	15	3	1	2-2-1	DT120	3-12-11-NA-211
S0198	Typhimurium	Human	0	0	0	NT	3.91E+21	1.11059E+13	4199	33-28-10	DT104	3-15-12-14-311
S0199	Typhimurium	Human	0	0	0	NT	19114095	32623493595	1	21-19-1	DT120	4-14-9-NA-211
S0200	1,4,[5],12:i:-	Human	0	0	0	A10	15	1	1	2-1-1	DT193	NA-12-9-NA-NA
S0201	1,4,[5],12:i:-	Human	0	0	0	NT	15	1	1	2-1-1	NT	3-12-10-NA-211
S0202	Typhimurium	Human	0	0	0	NT	19114095	1.40281E+12	1	21-25-1	DT116	3-13-8-NA-211
S0203	Typhimurium	Human	0	0	0	NT	255255	8843835	1155	16-12-8	DT193	3-16-10-NA-311
S0204	1,4,[5],12:i:-	Human	0	0	0	NT	15	1	1	2-1-1	DT193	3-12-10-NA-211
S0205	1,4,[5],12:i:-	Human	0	0	0	NT	15	1	1	2-1-1	DT120	3-11-11-NA-211
S0206	Typhimurium	Human	0	0	0	NT	255255	8843835	1155	16-12-8	DT193	3-15-12-NA-311
S0207	Typhimurium	Human	0	0	0	NT	345	57	29	6-4-2	DT135	2-11-11-12-NA
S0208	Typhimurium	Human	0	0	0	NT	19095	82203	1	12-6-1	DT7	3-13-10-NA-211
S0209	Typhimurium	Human	0	0	0	NT	10005	23339049	69	10-14-5	DT120	3-10-7-8-211
S0210	1,4,[5],12:i:-	Human	0	0	0	NT	19114095	10874497865	1	21-18-1	DT120	3-16-7-NA-211
S0211	1,4,[5],12:i:-	Human	0	0	0	NT	15	1	1	2-1-1	DT193	3-12-9-NA-NA
S0212	1,4,[5],12:i:-	Human	0	0	0	NT	15	1	1	2-1-1	DT193	3-12-10-NA-211

Sample_ID	Serovar	Source	Outbreak_related	PCR_screening	Stability_experiment	PFGE_cluster[a]	GPP_MOL-PCR_1	GPP_MOL-PCR_2	GPP_MOL-PCR_SNP	MOL-PCR_code	Phage_type[b]	MLVA
S0213	1,4,[5],12:i:-	Human	0	0	0	NT	15	1	1	2-1-1	DT193	3-12-10-NA-211
S0214	1,4,[5],12:i:-	Human	0	0	0	NT	15	1	1	2-1-1	DT120	3-12-10-NA-211
S0215	1,4,[5],12:i:-	Human	0	0	0	NT	15	1	1	2-1-1	DT120	3-13-5-NA-211
S0216	1,4,[5],12:i:-	Human	0	0	0	NT	15	1	1	2-1-1	DT120	3-12-9-NA-211
S0217	Typhimurium	Human	0	0	0	NT	255255	8843835	1155	16-12-8	DT120	3-14-11-NA-311
S0218	Typhimurium	Human	0	0	0	NT	255255	8843835	1155	16-12-8	DT193	3-16-10-NA-311
S0219	Typhimurium	Human	0	0	0	NT	15	3	1	2-2-1	DT193	3-12-11-NA-211
S0220	1,4,[5],12:i:-	Human	0	0	0	NT	15	1	1	2-1-1	DT110	3-12-10-NA-211
S0221	Typhimurium	Human	0	0	0	NT	345345	430422405	69	17-16-5	NT	4-9-10-11-211
S0222	Typhimurium	Human	0	0	0	NT	1.0028E+11	2.58277E+11	69	28-23-5	U311	4-14-15-7-NA
S0223	1,4,[5],12:i:-	Human	0	0	0	NT	15	1	1	2-1-1	DT120	3-13-14-NA-211
S0224	Typhimurium	Human	0	0	0	NT	19114095	32623493595	1	21-19-1	DT120	3-13-11-NA-NA
S0225	1,4,[5],12:i:-	Human	0	0	0	NT	15	1	1	2-1-1	DT120	3-12-9-NA-211
S0226	1,4,[5],12:i:-	Human	0	0	0	NT	19114095	10874497865	1	21-18-1	DT120	3-16-7-NA-211
S0227	1,4,[5],12:i:-	Human	0	0	0	NT	15	1	1	2-1-1	DT193	3-11-9-NA-211
S0228	Typhimurium	Human	0	0	0	A3	15	3	1	2-2-1	NT	3-12-11-NA-211
S0229	Typhimurium	Human	0	0	0	NT	255255	8843835	1155	16-12-8	NT	3-17-11-NA-311
S0230	1,4,[5],12:i:-	Human	0	0	0	NT	15	1	1	2-1-1	DT193	3-13-11-NA-211
S0231	Typhimurium	Human	0	0	0	NT	10005	23339049	69	10-14-5	RDNC	3-10-7-8-211
S0232	1,4,[5],12:i:-	Human	0	0	0	NT	15	1	1	2-1-1	DT138	3-12-11-NA-211
S0233	1,4,[5],12:i:-	Human	0	0	0	NT	15	1	1	2-1-1	DT193	3-13-9-NA-211
S0234	1,4,[5],12:i:-	Human	0	0	0	NT	15	1	1	2-1-1	DT193	3-13-8-NA-211
S0235	1,4,[5],12:i:-	Human	0	0	0	NT	15	1	1	2-1-1	DT193	3-11-9-NA-211
S0236	1,4,[5],12:i:-	Human	0	0	0	NT	15	1	1	2-1-1	DT138	3-12-11-NA-211
S0237	Typhimurium	Human	0	0	0	B3	255255	8843835	1155	16-12-8	DT193	3-NA-10-NA-NA
S0238	Typhimurium	Human	0	0	0	NT	255255	8843835	1155	16-12-8	DT193	3-17-11-NA-211
S0239	Typhimurium	Human	0	0	0	NT	255255	8843835	1155	16-12-8	DT193	3-15-10-NA-311
S0240	1,4,[5],12:i:-	Human	0	0	0	A10	15	1	1	2-1-1	DT193	3-13-11-NA-211
S0241	Typhimurium	Human	0	0	0	NT	345345	430422405	69	17-16-5	NT	4-9-11-10-NA
S0242	Typhimurium	Human	0	0	0	NT	3.91E+21	1.11059E+13	4199	33-28-10	DT104	3-14-13-22-NA
S0243	Typhimurium	Human	0	0	0	NT	15	3	1	2-2-1	DT120	3-12-11-NA-211
S0244	1,4,[5],12:i:-	Human	0	0	0	NT	15	1	1	2-1-1	DT193	3-12-9-NA-211
S0245	1,4,[5],12:i:-	Human	0	0	0	NT	15	1	1	2-1-1	DT193	3-14-10-NA-211
S0246	Typhimurium	Human	0	0	0	NT	3.91E+21	1.11059E+13	4199	33-28-10	DT104	3-12-NA-14-NA
S0247	Typhimurium	Human	0	0	0	NT	3.91E+21	1.11059E+13	4199	33-28-10	DT120	3-14-20-24-311
S0248	Typhimurium	Human	0	0	0	A11	15	3	1	2-2-1	DT120	3-12-11-NA-211
S0249	Typhimurium	Human	0	0	0	NT	9.54E+19	1.11059E+13	4199	31-28-10	DT104	3-14-11-25-211
S0250	Typhimurium	Human	0	0	0	NT	15	3	1	2-2-1	DT120	3-12-13-NA-211
S0251	1,4,[5],12:i:-	Human	0	0	0	NT	15	1	1	2-1-1	DT120	3-12-9-NA-211
S0252	1,4,[5],12:i:-	Human	0	0	0	NT	15	1	1	2-1-1	DT193	3-12-10-NA-211
S0253	1,4,[5],12:i:-	Human	0	0	0	NT	15	1	1	2-1-1	DT120	3-12-10-NA-211
S0254	1,4,[5],12:i:-	Human	0	0	0	NT	345	1	29	6-1-2	DT41	2-11-5-7-NA
S0255	1,4,[5],12:i:-	Human	0	0	1	NT	15	1	1	2-1-1	DT193	3-12-9-NA-211

Sample_ID	Serovar	Source	Outbreak_related	PCR_screening	Stability_experiment	PFGE_cluster[a]	GPP_MOL-PCR_1	GPP_MOL-PCR_2	GPP_MOL-PCR_SNP	MOL-PCR_code	Phage_type[b]	MLVA
S0256	Typhimurium	Human	0	0	0	NT	15	3	1	2-2-1	DT120	3-13-8-NA-211
S0257	Typhimurium	Human	0	0	0	NT	15	3	1	2-2-1	DT120	3-21-10-NA-211
S0258	Typhimurium	Human	0	0	0	A11	15	3	1	2-2-1	DT120	NA-13-13-NA-211
S0259	Typhimurium	Human	0	0	0	NT	15	3	1	2-2-1	DT110	3-13-9-NA-211
S0260	1,4,[5],12:i:-	Human	0	0	0	A10	15	1	1	2-1-1	DT193	3-13-10-NA-211
S0261	1,4,[5],12:i:-	Human	0	0	0	NT	15	1	1	2-1-1	DT193	3-21-13-NA-211
S0262	Typhimurium	Human	0	0	0	NT	255255	8843835	1155	16-12-8	DT193	3-16-10-NA-311
S0263	Typhimurium	Human	0	0	0	NT	15	3	1	2-2-1	DT195	2-14-10-NA-211
S0264	Typhimurium	Human	0	0	0	NT	15	3	1	2-2-1	DT120	3-13-12-NA-211
S0265	Typhimurium	Human	0	0	0	NT	255255	8843835	1155	16-12-8	DT193	3-17-11-NA-311
S0266	Typhimurium	Human	0	0	0	NT	3457939485	1.69216E+11	29	27-22-2	U302	2-14-6-12-212
S0267	Typhimurium	Human	0	0	0	NT	15	3	1	2-2-1	DT120	3-12-11-NA-211
S0268	1,4,[5],12:i:-	Human	0	0	0	NT	15	1	1	2-1-1	DT193	3-12-10-NA-211
S0269	Typhimurium	Human	0	0	0	NT	324939615	9.23053E+11	1	25-24-1	RDNC	3-17-7-NA-211
S0270	1,4,[5],12:i:-	Human	0	0	0	NT	15	1	1	2-1-1	DT120	3-13-9-NA-211
S0271	Typhimurium	Human	0	0	0	NT	15	3	1	2-2-1	DT120	3-9-9-NA-211
S0272	Typhimurium	Human	0	0	0	NT	15	3	1	2-2-1	DT120	3-12-11-NA-NA
S0273	Typhimurium	Human	0	0	0	NT	15	3	1	2-2-1	DT120	3-12-11-NA-211
S0274	Typhimurium	Human	0	0	0	NT	15	3	1	2-2-1	DT120	3-12-11-NA-NA
S0275	Typhimurium	Human	0	0	0	NT	15	3	1	2-2-1	DT120	3-9-9-NA-211
S0276	Typhimurium	Human	0	0	0	NT	255255	8843835	1155	16-12-8	DT208	3-14-12-NA-311
S0277	Typhimurium	Human	0	0	0	NT	255255	8843835	1155	16-12-8	DT193	3-15-12-NA-311
S0278	1,4,[5],12:i:-	Human	0	0	0	NT	15	1	1	2-1-1	DT110	3-13-9-NA-211
S0279	1,4,[5],12:i:-	Human	0	0	0	NT	15	1	1	2-1-1	DT120	3-14-8-NA-211
S0280	1,4,[5],12:i:-	Human	0	0	0	NT	15	1	1	2-1-1	DT110	3-12-10-NA-211
S0281	Typhimurium	Human	0	0	0	A11	15	3	1	2-2-1	DT120	3-9-9-NA-NA
S0282	Typhimurium	Human	0	0	0	B3	255255	8843835	1155	16-12-8	DT104	3-14-11-NA-NA
S0283	Typhimurium	Human	0	0	0	NT	15	3	1	2-2-1	DT120	3-9-9-NA-211
S0284	1,4,[5],12:i:-	Human	0	0	0	NT	15	1	1	2-1-1	DT193	3-12-10-NA-211
S0285	1,4,[5],12:i:-	Human	0	0	0	NT	15	1	1	2-1-1	DT138	3-13-9-NA-211
S0286	1,4,[5],12:i:-	Human	0	0	0	NT	15	1	1	2-1-1	DT193	3-14-9-NA-211
S0287	Typhimurium	Human	0	0	0	NT	2.04963E+16	1.11059E+13	4199	29-28-10	DT120	3-15-5-12-311
S0288	Typhimurium	Human	0	0	0	NT	15	3	1	2-2-1	DT120	3-12-11-NA-211
S0289	1,4,[5],12:i:-	Human	0	0	0	A1	15	1	1	2-1-1	DT56	4-12-9-NA-211
S0290	1,4,[5],12:i:-	Human	0	0	0	NT	19114095	10874497865	1	21-18-1	DT120	3-14-7-NA-211
S0291	Typhimurium	Human	0	0	0	NT	1.0028E+11	2.58277E+11	69	28-23-5	DT193	4-14-8-7-211
S0292	Typhimurium	Human	0	0	0	NT	15	3	1	2-2-1	DT120	3-13-8-NA-211
S0293	Typhimurium	Human	0	0	0	NT	15	3	1	2-2-1	DT120	3-9-9-NA-211
S0294	Typhimurium	Human	0	0	0	NT	15	3	1	2-2-1	DT120	3-13-8-NA-211
S0295	Typhimurium	Human	0	0	0	NT	15	3	1	2-2-1	NT	3-13-12-NA-211
S0296	Typhimurium	Human	0	0	0	NT	345	23339049	29	6-14-2	RDNC	2-13-26-13-212
S0297	Typhimurium	Human	0	0	0	NT	15	3	1	2-2-1	DT120	3-12-11-NA-211
S0298	1,4,[5],12:i:-	Human	0	0	0	NT	15	1	1	2-1-1	DT120	3-13-10-NA-211

Sample_ID	Serovar	Source	Outbreak_related	PCR_screening	Stability_experiment	PFGE_cluster[a]	GPP_MOL-PCR_1	GPP_MOL-PCR_2	GPP_MOL-PCR_SNP	MOL-PCR_code	Phage_type[b]	MLVA
S0299	Typhimurium	Human	0	0	0	NT	87	3	105	4-2-6	RDNC	3-16-2-NA-311
S0300	Typhimurium	Human	0	0	0	NT	19114095	32623493595	1	21-19-1	DT193	3-13-8-NA-211
S0301	Typhimurium	Human	0	0	0	NT	15	3	1	2-2-1	DT120	3-9-9-NA-211
S0302	1,4,[5],12:i:-	Human	0	0	0	NT	15	1	1	2-1-1	DT193	3-13-8-NA-211
S0303	Typhimurium	Human	0	0	0	NT	15	3	1	2-2-1	DT120	3-9-9-NA-211
S0304	Typhimurium	Human	0	0	0	NT	255255	8843835	1155	16-12-8	DT193	3-11-11-NA-311
S0305	Typhimurium	Human	0	0	0	NT	19114095	32623493595	1	21-19-1	RDNC	3-12-8-NA-211
S0306	1,4,[5],12:i:-	Human	0	0	0	NT	15	1	1	2-1-1	DT120	3-13-7-NA-211
S0307	1,4,[5],12:i:-	Human	0	0	0	NT	15	1	1	2-1-1	DT193	3-13-8-NA-211
S0308	1,4,[5],12:i:-	Human	0	0	0	NT	15	1	1	2-1-1	DT120	3-12-9-NA-211
S0309	1,4,[5],12:i:-	Human	0	0	0	NT	15	1	1	2-1-1	DT193	3-13-9-NA-211
S0310	1,4,[5],12:i:-	Human	0	0	0	NT	795	1	1	8-1-1	DT193	3-13-11-NA-211
S0311	1,4,[5],12:i:-	Human	0	0	0	A1	15	1	1	2-1-1	RDNC	4-12-9-NA-211
S0312	1,4,[5],12:i:-	Human	0	0	0	NT	15	1	1	2-1-1	DT120	3-13-10-NA-211
S0313	Typhimurium	Human	0	0	0	NT	19114095	1.40281E+12	1	21-25-1	DT193	3-13-9-NA-211
S0314	Typhimurium	Human	0	0	0	NT	19114095	1.40281E+12	1	21-25-1	DT120	3-13-9-NA-211
S0315	1,4,[5],12:i:-	Human	0	0	0	NT	15	1	1	2-1-1	DT193	3-13-8-NA-211
S0316	Typhimurium	Human	0	0	0	NT	15	3	1	2-2-1	DT120	3-9-9-NA-211
S0317	1,4,[5],12:i:-	Human	0	0	0	NT	795	1	1	8-1-1	DT193	3-8-10-NA-211
S0318	Typhimurium	Human	0	0	0	NT	3.91E+21	1.11059E+13	4199	33-28-10	DT110	3-15-16-14-311
S0319	1,4,[5],12:i:-	Human	0	0	0	NT	15	1	1	2-1-1	RDNC	3-12-11-NA-211
S0320	1,4,[5],12:i:-	Human	0	0	0	NT	15	1	1	2-1-1	RDNC	3-12-11-NA-211
S0321	1,4,[5],12:i:-	Human	0	0	0	NT	19114095	10874497865	1	21-18-1	DT120	3-14-7-NA-211
S0322	Typhimurium	Human	0	0	0	NT	23138115	4.50435E+17	69	22-30-5	DT195	3-10-11-7-211
S0323	Typhimurium	Human	0	0	0	NT	150345195	4.90726E+12	1155	24-27-8	DT193	3-15-12-NA-311
S0324	Typhimurium	Human	0	0	0	NT	15	3	1	2-2-1	DT120	3-9-9-NA-211
S0325	1,4,[5],12:i:-	Human	0	0	0	NT	15	1	1	2-1-1	DT120	3-12-9-NA-211
S0326	1,4,[5],12:i:-	Human	0	0	0	NT	15	1	1	2-1-1	DT193	3-13-8-NA-211
S0327	Typhimurium	Human	0	0	0	NT	1312311	291165	29	18-8-2	DT12	2-21-17-8-212
S0328	1,4,[5],12:i:-	Human	0	0	0	NT	15	1	1	2-1-1	DT193	3-13-8-NA-211
S0329	Typhimurium	Human	0	0	0	NT	5870865	8843835	69	19-12-5	DT35	3-12-10-9-211
S0330	1,4,[5],12:i:-	Human	0	0	0	NT	15	1	1	2-1-1	RDNC	3-12-11-NA-211
S0331	Typhimurium	Human	0	0	0	NT	3.91E+21	1.11059E+13	4199	33-28-10	DT120	3-13-15-23-311
S0332	1,4,[5],12:i:-	Human	0	0	0	NT	15	1	1	2-1-1	DT193	3-13-8-NA-211
S0333	Typhimurium	Human	0	0	0	A14	15	3	1	2-2-1	DT135	3-17-9-NA-211
S0334	Typhimurium	Human	0	0	0	NT	19114095	1.40281E+12	1	21-25-1	DT193	3-13-9-NA-211
S0335	1,4,[5],12:i:-	Human	0	0	0	NT	15	1	1	2-1-1	DT193	3-13-9-NA-211
S0336	Typhimurium	Human	0	0	0	NT	3457939485	1.69216E+11	29	27-22-2	NT	2-14-7-12-212
S0337	1,4,[5],12:i:-	Human	0	0	1	NT	15	1	1	2-1-1	DT193	3-13-8-NA-211
S0338	1,4,[5],12:i:-	Human	0	0	0	NT	15	1	1	2-1-1	DT193	3-11-9-NA-211
S0339	1,4,[5],12:i:-	Human	0	0	1	NT	15	1	1	2-1-1	DT193	3-12-10-NA-211
S0340	Typhimurium	Human	0	0	0	NT	345	3	41	6-2-4	DT44	2-11-11-10-212
S0341	1,4,[5],12:i:-	Human	0	0	0	A8	15	1	1	2-1-1	DT120	3-12-9-NA-211

Sample_ID	Serovar	Source	Outbreak_related	PCR_screening	Stability_experiment	PFGE_cluster[a]	GPP_MOL-PCR_1	GPP_MOL-PCR_2	GPP_MOL-PCR_SNP	MOL-PCR_code	Phage_type[b]	MLVA
S0342	Typhimurium	Human	0	0	0	NT	255255	8843835	1155	16-12-8	DT193	3-15-13-NA-311
S0343	1,4,[5],12:i:-	Human	0	0	0	A6	15	1	1	2-1-1	NT	3-13-10-NA-211
S0344	Typhimurium	Human	0	0	0	NT	851	3	221	9-2-7	DT1	3-8-6-11-311
S0345	Typhimurium	Human	0	0	0	NT	1.0028E+11	2.58277E+11	69	28-23-5	U311	4-13-13-7-211
S0346	Typhimurium	Human	0	0	0	NT	15	3	1	2-2-1	DT120	3-12-10-NA-211
S0347	Typhimurium	Human	0	0	0	NT	15	3	1	2-2-1	DT120	3-9-9-NA-211
S0348	1,4,[5],12:i:-	Human	0	0	0	NT	15	1	1	2-1-1	NT	3-13-10-NA-211
S0349	Typhimurium	Human	0	0	0	NT	255255	8843835	1155	16-12-8	DT193	3-15-12-NA-311
S0350	Typhimurium	Human	0	0	0	NT	574893165	1.54007E+11	29	26-21-2	DT193	2-21-17-12-212
S0351	1,4,[5],12:i:-	Human	0	0	0	NT	15	1	1	2-1-1	NT	3-13-10-NA-211
S0352	Typhimurium	Human	0	0	0	NT	1.0028E+11	2.58277E+11	69	28-23-5	NT	4-13-13-7-211
S0353	Typhimurium	Human	0	0	0	NT	1.0028E+11	2.58277E+11	69	28-23-5	DT120	4-13-8-7-211
S0354	Typhimurium	Human	0	0	0	NT	851	1228371	221	9-11-7	RDNC	3-9-8-25-312
S0355	1,4,[5],12:i:-	Human	0	0	0	NT	15	1	1	2-1-1	DT193	3-13-8-NA-211
S0356	Typhimurium	Human	0	0	0	NT	15	3	1	2-2-1	DT193	3-13-10-NA-211
S0357	Typhimurium	Human	0	0	0	NT	15	3	1	2-2-1	DT193	3-13-10-NA-211
S0358	Typhimurium	Human	0	0	0	NT	345345	8178025695	29	17-17-2	DT15	2-11-8-9-212
S0359	Typhimurium	Human	0	0	0	NT	15	3	1	2-2-1	DT120	3-9-9-NA-211
S0360	1,4,[5],12:i:-	Human	0	0	0	NT	15	1	1	2-1-1	DT193	3-11-9-NA-211
S0361	1,4,[5],12:i:-	Human	0	0	0	NT	15	1	1	2-1-1	DT138	3-12-11-NA-211
S0362	Typhimurium	Human	0	0	0	NT	15	3	1	2-2-1	DT120	3-9-9-NA-211
S0363	Typhimurium	Human	0	0	0	NT	851	3	221	9-2-7	DT1	3-13-13-12-312
S0364	1,4,[5],12:i:-	Human	0	0	0	NT	15	1	1	2-1-1	DT138	3-12-11-NA-211
S0365	Typhimurium	Human	0	0	0	NT	255255	8843835	1155	16-12-8	DT12	3-14-12-NA-311
S0366	Typhimurium	Human	0	0	0	NT	2.04963E+16	1.11059E+13	4199	29-28-10	DT12	3-14-5-18-311
S0367	Typhimurium	Human	0	0	0	NT	255255	8843835	1155	16-12-8	DT193	3-14-12-NA-311
S0368	Typhimurium	Human	0	0	0	NT	15	3	1	2-2-1	DT120	3-13-8-NA-211
S0369	Typhimurium	Human	0	0	0	NT	255255	8843835	1155	16-12-8	DT193	3-15-12-NA-311
S0370	1,4,[5],12:i:-	Human	0	0	0	NT	15	1	1	2-1-1	DT120	3-12-9-NA-211
S0371	Typhimurium	Human	0	0	0	NT	435	57	1155	7-4-8	DT99	3-14-16-NA-311
S0372	Typhimurium	Human	0	0	0	NT	435	57	1155	7-4-8	DT96	3-14-16-NA-311
S0373	Typhimurium	Human	0	0	0	A7	15	3	1	2-2-1	DT7	3-12-10-NA-211
S0374	1,4,[5],12:i:-	Human	0	0	0	NT	15	1	1	2-1-1	DT138	3-12-11-NA-211
S0375	Typhimurium	Human	0	0	0	NT	851	3	221	9-2-7	DT1	6-12-8-10-311
S0376	Typhimurium	Human	0	0	0	NT	574893165	1.54007E+11	29	26-21-2	NT	2-21-17-12-212
S0377	Typhimurium	Human	0	0	0	NT	255255	8843835	1155	16-12-8	DT193	3-12-10-NA-311
S0378	Typhimurium	Human	0	0	0	B3	255255	8843835	1155	16-12-8	DT41	3-15-12-NA-311
S0379	Typhimurium	Human	0	0	0	NT	255255	8843835	1155	16-12-8	DT193	3-15-12-NA-311
S0380	1,4,[5],12:i:-	Human	0	0	0	NT	345	1	29	6-1-2	DT193	2-11-6-9-212
S0381	Typhimurium	Human	0	0	0	NT	255255	8843835	1155	16-12-8	DT193	3-12-10-NA-311
S0382	1,4,[5],12:i:-	Human	0	0	0	NT	19114095	10874497865	1	21-18-1	DT120	3-14-8-NA-211
S0383	Typhimurium	Human	0	0	0	NT	574893165	1.54007E+11	29	26-21-2	DT193	2-21-18-12-212
S0384	1,4,[5],12:i:-	Human	0	0	0	NT	15	1	1	2-1-1	DT193	3-13-10-NA-211

Sample_ID	Serovar	Source	Outbreak_related	PCR_screening	Stability_experiment	PFGE_cluster[a]	GPP_MOL-PCR_1	GPP_MOL-PCR_2	GPP_MOL-PCR_SNP	MOL-PCR_code	Phage_type[b]	MLVA
S0385	Typhimurium	Human	0	0	0	NT	3.91E+21	1.11059E+13	180557	33-28-11	DT104	3-15-10-14-311
S0386	Typhimurium	Human	0	0	0	NT	15	3	1	2-2-1	DT120	3-9-9-NA-211
S0387	Typhimurium	Human	0	0	0	NT	15	3	1	2-2-1	DT120	3-9-9-NA-211
S0388	1,4,[5],12:i:-	Human	0	0	0	A10	15	1	1	2-1-1	DT193	3-11-8-NA-111
S0389	1,4,[5],12:i:-	Human	0	0	0	NT	15	1	1	2-1-1	DT138	3-12-11-NA-211
S0390	Typhimurium	Human	0	0	0	NT	345	57	29	6-4-2	DT2	2-10-11-12-312
S0391	Typhimurium	Human	0	0	0	NT	2.04963E+16	1.11059E+13	4199	29-28-10	U302	3-14-5-18-311
S0392	Typhimurium	Human	0	0	0	NT	1.20928E+18	2.58277E+11	4199	30-23-10	DT104	3-16-16-13-311
S0393	1,4,[5],12:i:-	Human	0	0	0	NT	15	1	1	2-1-1	DT138	3-11-12-NA-211
S0394	1,4,[5],12:i:-	Human	0	0	0	NT	15	1	1	2-1-1	DT138	3-12-11-NA-211
S0395	Typhimurium	Human	0	0	0	NT	10005	23339049	69	10-14-5	RDNC	3-10-7-8-211
S0396	Typhimurium	Human	0	0	0	NT	255255	8843835	1155	16-12-8	DT193	3-15-13-NA-311
S0397	Typhimurium	Human	0	0	0	NT	1.0028E+11	2.58277E+11	69	28-23-5	U311	3-13-8-7-211
S0398	1,4,[5],12:i:-	Human	0	0	0	NT	15	1	1	2-1-1	DT193	3-12-9-NA-211
S0399	1,4,[5],12:i:-	Human	0	0	0	NT	15	1	1	2-1-1	DT120	3-12-9-NA-211
S0400	1,4,[5],12:i:-	Human	0	0	0	NT	345	1	29	6-1-2	DT41	2-11-6-9-212
S0401	Typhimurium	Human	0	0	0	NT	19114095	32623493595	1	21-19-1	DT120	3-13-11-NA-211
S0402	1,4,[5],12:i:-	Human	0	0	0	NT	15	1	1	2-1-1	DT138	3-12-11-NA-211
S0403	Typhimurium	Human	0	0	0	NT	1.0028E+11	2.58277E+11	69	28-23-5	DT120	4-13-8-7-211
S0404	Typhimurium	Human	0	0	0	NT	15	3	1	2-2-1	DT120	3-9-9-NA-211
S0405	Typhimurium	Human	0	0	0	NT	15	3	1	2-2-1	DT120	3-12-11-NA-211
S0406	Typhimurium	Human	0	0	0	NT	15	3	1	2-2-1	DT138	3-14-9-NA-211
S0407	Typhimurium	Human	0	0	0	NT	3.91E+21	1.11059E+13	4199	33-28-10	DT120	3-13-18-13-311
S0408	1,4,[5],12:i:-	Human	0	0	0	NT	15	1	1	2-1-1	DT138	3-12-11-NA-211
S0409	1,4,[5],12:i:-	Human	0	0	0	NT	15	1	1	2-1-1	DT138	3-12-11-NA-211
S0410	Typhimurium	Human	0	0	0	NT	345	23339049	29	6-14-2	DT2	2-10-11-12-312
S0411	Typhimurium	Human	0	0	0	NT	1.0028E+11	2.58277E+11	69	28-23-5	RDNC	4-13-18-7-211
S0412	Typhimurium	Human	0	0	0	NT	15	3	1	2-2-1	DT120	3-14-8-NA-211
S0413	Typhimurium	Human	0	0	0	NT	3.91E+21	1.11059E+13	4199	33-28-10	DT104	3-12-20-14-311
S0414	Typhimurium	Human	0	0	0	NT	15	3	1	2-2-1	DT120	3-9-9-NA-211
S0415	1,4,[5],12:i:-	Human	0	0	0	NT	15	17606651	1	2-13-1	DT120	3-12-11-NA-211
S0416	Typhimurium	Human	0	0	0	NT	15	3	1	2-2-1	DT120	3-12-9-NA-211
S0417	Typhimurium	Human	0	0	0	NT	574893165	1.54007E+11	29	26-21-2	NT	2-21-17-11-212
S0418	Typhimurium	Human	0	0	0	B3	255255	8843835	1155	16-12-8	DT120	3-15-12-NA-311
S0419	Typhimurium	Human	0	0	0	NT	15	3	1	2-2-1	DT193	3-12-10-NA-211
S0420	1,4,[5],12:i:-	Human	0	0	0	NT	15	1	1	2-1-1	DT120	3-12-9-NA-211
S0421	1,4,[5],12:i:-	Human	0	0	0	NT	15	1	1	2-1-1	DT138	3-13-10-NA-211
S0422	1,4,[5],12:i:-	Human	0	0	0	NT	15	1	1	2-1-1	DT120	3-12-9-NA-211
S0423	1,4,[5],12:i:-	Human	0	0	0	NT	15	1	1	2-1-1	DT138	3-11-12-NA-211
S0424	Typhimurium	Human	0	0	0	NT	19114095	32623493595	1	21-19-1	RDNC	3-12-8-NA-211
S0425	1,4,[5],12:i:-	Human	0	0	0	NT	15	1	1	2-1-1	DT120	3-13-9-NA-211
S0426	Typhimurium	Human	0	0	0	NT	3.91E+21	1.11059E+13	4199	33-28-10	DT104	3-13-9-17-311
S0427	1,4,[5],12:i:-	Human	0	0	0	NT	15	1	1	2-1-1	DT193	3-12-8-NA-211

Sample_ID	Serovar	Source	Outbreak_related	PCR_screening	Stability_experiment	PFGE_cluster[a]	GPP_MOL-PCR_1	GPP_MOL-PCR_2	GPP_MOL-PCR_SNP	MOL-PCR_code	Phage_type[b]	MLVA
S0428	Typhimurium	Human	0	0	0	B1	255255	8843835	1155	16-12-8	DT12	3-10-7-NA-311
S0429	1,4,[5],12:i:-	Human	0	0	0	NT	19114095	10874497865	1	21-18-1	DT120	3-14-8-NA-211
S0430	Typhimurium	Human	0	0	0	NT	1.0028E+11	2.58277E+11	69	28-23-5	RDNC	4-13-18-7-211
S0431	1,4,[5],12:i:-	Human	0	0	0	NT	19114095	10874497865	1	21-18-1	DT120	3-14-8-NA-211
S0432	1,4,[5],12:i:-	Human	0	0	0	NT	15	1	1	2-1-1	DT193	3-12-8-NA-211
S0433	Typhimurium	Human	0	0	0	NT	15	3	1	2-2-1	DT120	3-12-10-NA-211
S0434	1,4,[5],12:i:-	Human	0	0	0	NT	19114095	10874497865	1	21-18-1	DT120	3-14-8-NA-211
S0435	Typhimurium	Human	0	0	0	NT	15	3	1	2-2-1	DT120	3-9-9-NA-211
S0436	Typhimurium	Human	0	0	0	NT	255255	8843835	1155	16-12-8	DT194	3-15-10-NA-311
S0437	1,4,[5],12:i:-	Human	0	0	0	NT	15	1	1	2-1-1	DT193	3-13-10-NA-211
S0438	1,4,[5],12:i:-	Human	0	0	0	NT	15	1	1	2-1-1	DT120	3-12-9-NA-211
S0439	1,4,[5],12:i:-	Human	0	0	0	NT	23055	43	1	13-3-1	NT	3-13-7-NA-111
S0440	1,4,[5],12:i:-	Human	0	0	0	NT	15	1	1	2-1-1	DT193	3-14-9-NA-211
S0441	Typhimurium	Human	0	0	0	NT	255255	8843835	1155	16-12-8	DT194	3-11-12-NA-311
S0442	Typhimurium	Human	0	0	0	NT	324939615	9.23053E+11	1	25-24-1	RDNC	3-17-9-NA-211
S0443	Typhimurium	Human	0	0	0	NT	3.91E+21	1.11059E+13	4199	33-28-10	DT12	3-15-16-14-311
S0444	Typhimurium	Human	0	0	0	NT	3.91E+21	1.11059E+13	4199	33-28-10	U302	3-15-16-14-311
S0445	Typhimurium	Human	0	0	0	NT	19114095	1.40281E+12	1	21-25-1	DT194	3-13-8-NA-211
S0446	Typhimurium	Human	0	0	0	NT	15	3	1	2-2-1	DT120	3-12-10-NA-211
S0447	1,4,[5],12:i:-	Human	0	0	0	NT	15	1	1	2-1-1	RDNC	3-13-14-NA-211
S0448	Typhimurium	Human	0	0	0	NT	255255	8843835	1155	16-12-8	U302	3-15-10-NA-311
S0449	1,4,[5],12:i:-	Human	0	0	0	NT	345	1	29	6-1-2	RDNC	2-11-5-7-212
S0450	1,4,[5],12:i:-	Human	0	0	0	NT	15	1	1	2-1-1	DT193	3-12-8-NA-211
S0451	Typhimurium	Human	0	0	0	NT	255255	8843835	1155	16-12-8	U302	3-11-11-NA-311
S0452	1,4,[5],12:i:-	Human	0	0	0	NT	19114095	10874497865	1	21-18-1	DT120	3-14-8-NA-211
S0453	Typhimurium	Human	0	0	0	NT	255255	8843835	1155	16-12-8	DT193	3-17-11-NA-311
S0454	1,4,[5],12:i:-	Human	0	0	0	NT	15	1	1	2-1-1	DT120	3-15-10-NA-211
S0455	Typhimurium	Human	0	0	0	NT	15	3	1	2-2-1	DT120	3-12-10-NA-211
S0456	Typhimurium	Human	0	0	0	NT	345345	430422405	69	17-16-5	DT195	4-9-8-12-211
S0457	Typhimurium	Human	0	0	0	NT	15	129	1	2-5-1	DT195	3-12-12-NA-211
S0458	1,4,[5],12:i:-	Human	0	0	0	NT	15	1	1	2-1-1	DT193	3-14-9-NA-211
S0459	Typhimurium	Human	0	0	0	B3	255255	8843835	1155	16-12-8	DT194	3-15-12-NA-311
S0460	Typhimurium	Human	0	0	0	NT	345345	8178025695	29	17-17-2	RDNC	2-12-8-9-212
S0461	Typhimurium	Human	0	0	0	NT	15	57	1155	2-4-8	DT193	3-17-11-NA-211
S0462	Typhimurium	Human	0	0	0	NT	2.04963E+16	1.11059E+13	4199	29-28-10	DT104	3-14-15-22-311
S0463	Typhimurium	Human	0	0	0	NT	255255	8843835	1155	16-12-8	NT	3-11-11-NA-311
S0464	Typhimurium	Human	0	0	0	NT	3.91E+21	1.11059E+13	4199	33-28-10	DT104	3-13-12-22-311
S0465	1,4,[5],12:i:-	Human	0	0	0	A10	15	1	1	2-1-1	DT194	3-15-11-NA-211
S0466	Typhimurium	Human	0	0	0	NT	345345	8178025695	29	17-17-2	RDNC	2-12-8-9-212
S0467	Typhimurium	Human	0	0	0	NT	15	129	1	2-5-1	NT	3-12-12-NA-211
S0468	1,4,[5],12:i:-	Human	0	0	0	A7	15	1	1	2-1-1	U311	3-13-11-NA-211
S0469	Typhimurium	Human	0	0	0	NT	15	3	1	2-2-1	DT120	3-13-12-NA-211
S0470	Typhimurium	Human	0	0	0	NT	345345	1.76239E+14	69	17-29-5	U310	4-10-12-9-211

Sample_ID	Serovar	Source	Outbreak_related	PCR_screening	Stability_experiment	PFGE_cluster[a]	GPP_MOL-PCR_1	GPP_MOL-PCR_2	GPP_MOL-PCR_SNP	MOL-PCR_code	Phage_type[b]	MLVA
S0471	1,4,[5],12:i:-	Human	0	0	0	NT	19114095	10874497865	1	21-18-1	DT120	3-13-9-NA-211
S0472	Typhimurium	Human	0	0	0	NT	255255	8843835	1155	16-12-8	DT193	3-15-10-NA-311
S0473	Typhimurium	Human	0	0	0	NT	15	3	1	2-2-1	DT120	3-13-9-NA-211
S0474	1,4,[5],12:i:-	Human	0	0	0	A1	15	1	1	2-1-1	DT120	4-12-9-NA-211
S0475	Typhimurium	Human	0	0	0	A15	15	3	1	2-2-1	DT138	3-12-10-NA-211
S0476	1,4,[5],12:i:-	Human	0	0	0	NT	15	1	1	2-1-1	DT120	3-13-10-NA-211
S0477	Typhimurium	Human	0	0	0	NT	255255	8843835	1155	16-12-8	DT194	3-11-11-NA-311
S0478	Typhimurium	Human	0	0	0	NT	255255	8843835	1155	16-12-8	DT194	3-17-11-NA-311
S0479	Typhimurium	Human	0	0	0	NT	3.91E+21	1.11059E+13	4199	33-28-10	DT104	3-14-14-22-311
S0480	1,4,[5],12:i:-	Human	0	0	0	NT	15	1	1	2-1-1	DT120	3-12-11-NA-211
S0481	Typhimurium	Human	0	0	0	NT	851	3	221	9-2-7	DT1	3-15-9-14-311
S0482	Typhimurium	Human	0	0	0	NT	15	3	1	2-2-1	DT120	3-13-12-NA-211
S0483	Typhimurium	Human	0	0	0	NT	255255	8843835	1155	16-12-8	DT193	3-17-11-NA-311
S0484	Typhimurium	Human	0	0	0	NT	255255	8843835	1155	16-12-8	DT193	3-11-12-NA-311
S0485	Typhimurium	Human	0	0	0	NT	255255	8843835	1155	16-12-8	DT193	3-11-12-NA-311
S0486	Typhimurium	Human	0	0	0	NT	255255	8843835	1155	16-12-8	DT193	3-11-12-NA-311
S0487	1,4,[5],12:i:-	Human	0	0	0	NT	15	1	1	2-1-1	DT193	3-13-10-NA-211
S0488	1,4,[5],12:i:-	Human	0	0	0	A12	15	1	1	2-1-1	NT	3-13-11-NA-211
S0489	Typhimurium	Human	0	0	0	NT	255255	8843835	1155	16-12-8	DT193	3-17-11-NA-311
S0490	Typhimurium	Human	0	0	0	NT	255255	8843835	1155	16-12-8	DT193	3-15-11-NA-311
S0491	Typhimurium	Human	0	0	0	NT	255255	8843835	1155	16-12-8	DT193	3-15-11-NA-311
S0492	Typhimurium	Human	0	0	0	NT	2.04963E+16	1.11059E+13	4199	29-28-10	U302	3-14-5-18-311
S0493	Typhimurium	Human	0	0	0	NT	851	1228371	221	9-11-7	DT41	3-9-8-25-312
S0494	Typhimurium	Human	0	0	0	NT	1.06E+20	1.11059E+13	4199	32-28-10	U302	3-14-NA-12-311
S0495	Typhimurium	Human	0	0	0	NT	345	57	29	6-4-2	DT2	2-11-10-10-312
S0496	1,4,[5],12:i:-	Human	0	0	0	NT	15	1	1	2-1-1	NT	3-12-10-NA-211
S0497	Typhimurium	Human	0	0	0	NT	324939615	9.23053E+11	1	25-24-1	RDNC	3-18-9-NA-211
S0498	Typhimurium	Human	0	0	0	NT	255255	8843835	1155	16-12-8	DT193	3-15-11-NA-311
S0499	Typhimurium	Human	0	0	0	NT	255255	8843835	1155	16-12-8	DT193	3-14-12-NA-311
S0500	Typhimurium	Human	0	0	0	NT	15	3	1	2-2-1	DT120	3-12-10-NA-211
S0501	1,4,[5],12:i:-	Human	0	0	0	A16	15	1	1	2-1-1	NT	3-14-9-NA-111
S0502	Typhimurium	Human	0	0	0	NT	255255	8843835	1155	16-12-8	DT193	3-17-11-NA-311
S0503	Typhimurium	Human	0	0	0	NT	3.91E+21	1.11059E+13	4199	33-28-10	U302	3-16-13-4-311
S0504	Typhimurium	Human	0	0	0	B4	255255	8843835	1155	16-12-8	DT185	3-12-9-NA-211
S0505	Typhimurium	Human	0	0	0	NT	255255	8843835	1155	16-12-8	DT193	3-15-10-NA-311
S0506	Typhimurium	Human	0	0	0	NT	15	3	1	2-2-1	DT120	3-12-9-NA-211
S0507	Typhimurium	Human	0	0	0	NT	255255	8843835	1155	16-12-8	DT193	3-12-10-NA-311
S0508	Typhimurium	Human	0	0	0	NT	345345	430422405	69	17-16-5	NT	4-9-19-9-211
S0509	1,4,[5],12:i:-	Human	0	0	0	NT	15	1	1	2-1-1	DT193	3-12-8-NA-211
S0510	Typhimurium	Human	0	0	0	NT	255255	8843835	1155	16-12-8	DT193	3-14-11-NA-311
S0511	Typhimurium	Human	0	0	0	NT	255255	8843835	1155	16-12-8	DT193	3-14-11-NA-311
S0512	Typhimurium	Human	0	0	0	NT	15	3	1	2-2-1	DT120	3-13-12-NA-211
S0513	Typhimurium	Human	0	0	0	NT	255255	8843835	1155	16-12-8	DT193	3-14-11-NA-311

Sample_ID	Serovar	Source	Outbreak_related	PCR_screening	Stability_experiment	PFGE_cluster[a]	GPP_MOL-PCR_1	GPP_MOL-PCR_2	GPP_MOL-PCR_SNP	MOL-PCR_code	Phage_type[b]	MLVA
S0514	Typhimurium	Human	0	0	0	NT	3.91E+21	1.11059E+13	4199	33-28-10	DT104	3-12-16-22-311
S0515	Typhimurium	Human	0	0	0	NT	345345	8178025695	29	17-17-2	RDNC	2-13-8-9-212
S0516	Typhimurium	Human	0	0	0	NT	255255	8843835	1155	16-12-8	NT	3-14-11-NA-311
S0517	Typhimurium	Human	0	0	0	NT	255255	8843835	1155	16-12-8	NT	3-14-11-NA-311
S0518	Typhimurium	Human	0	0	0	NT	15	3	1	2-2-1	NT	3-15-9-NA-211
S0519	Typhimurium	Human	0	0	0	B3	255255	8843835	1155	16-12-8	NT	3-15-11-NA-311
S0520	Typhimurium	Human	1	0	0	NT	19114095	32623493595	1	21-19-1	DT195	3-12-10-NA-311
S0521	Typhimurium	Human	1	0	0	NT	19114095	32623493595	1	21-19-1	DT195	3-12-10-NA-311
S0522	Typhimurium	Human	1	0	0	NT	19114095	32623493595	1	21-19-1	DT195	3-12-10-NA-311
S0523	Typhimurium	Human	1	0	0	NT	19114095	32623493595	1	21-19-1	DT195	3-12-10-NA-311
S0524	Typhimurium	Human	1	0	0	NT	19114095	32623493595	1	21-19-1	DT195	3-12-10-NA-311
S0525	Typhimurium	Human	1	0	0	NT	15	3	1	2-2-1	DT120	3-15-5-NA-211
S0526	1,4,[5],12:i:-	Human	1	0	0	NT	15	1	1	2-1-1	DT138	3-13-11-NA-211
S0527	1,4,[5],12:i:-	Human	1	0	0	NT	15	1	1	2-1-1	DT138	3-13-11-NA-211
S0528	1,4,[5],12:i:-	Human	1	0	0	NT	15	1	1	2-1-1	DT138	3-13-11-NA-211
S0529	1,4,[5],12:i:-	Human	1	0	0	NT	15	1	1	2-1-1	DT138	3-13-11-NA-211
S0530	1,4,[5],12:i:-	Human	1	0	0	NT	15	1	1	2-1-1	DT138	3-13-11-NA-211
S0531	Typhimurium	Human	1	0	0	NT	15	3	1	2-2-1	RDNC	3-14-11-NA-211
S0532	Typhimurium	Human	1	0	1	NT	1.20928E+18	2.58277E+11	4199	30-23-10	DT104	3-14-18-14-311

[a] NT: not tested

[b] NT: not-typable; RDNC: reacts-but-does–not-conform

References

Barbau-Piednoir E, Bertrand S, Mahillon J, Roosens NH, Botteldoorn N (2013) SYBR®Green qPCR *Salmonella* detection system allowing discrimination at the genus, species and subspecies levels. Appl Microbiol Biotechnol 97:9811-9824. doi:10.1007/s00253-013-5234-x

Boyd D, Cloeckaert A, Chaslus-Dancla E, Mulvey MR (2002) Characterization of variant *Salmonella* genomic island 1 multidrug resistance regions from serovars Typhimurium DT104 and Agona. Antimicrob Agents Chemother 46:1714-1722. doi:10.1128/AAC.46.6.1714-1722.2002

Boyd D, Peters GA, Cloeckaert A, Boumedine KS, Chaslus-Dancla E, Imberechts H, Mulvey MR (2001) Complete nucleotide sequence of a 43-kilobase genomic island associated with the multidrug resistance region of *Salmonella enterica* serovar Typhimurium DT104 and its identification in phage type DT120 and serovar Agona. J Bacteriol 183:5725-5732. doi:10.1128/JB.183.19.5725-5732.2001

Boyd DA, Peters GA, Ng L-K, Mulvey MR (2000) Partial characterization of a genomic island associated with the multidrug resistance region of *Salmonella enterica* Typhymurium DT104. FEMS Microbiol Lett 189:285-291. doi:S0378109700002640 [pii]

Drahovská H, Mikasová E, Szemes T, Ficek A, Sásik M, Majtán V, Turna J (2007) Variability in occurrence of multiple prophage genes in *Salmonella* Typhimurium strains isolated in Slovak Republic. FEMS Microbiol Lett 270:237-244. doi:10.1111/j.1574-6968.2007.00674.x

Fang N-X, Huang B, Hiley L, Bates J, Savill J (2012) A rapid multiplex DNA suspension array method for *Salmonella typhimurium* subtyping using prophage-related markers. J Microbiol Methods 88:19-27. doi:10.1016/j.mimet.2011.10.002

Hernández Guijarro K, Feingold SE, Terzolo HR (2012) A single nucleotide polymorphism on *rpoB* gene allows specific identification of *Salmonella enterica* serotype Typhimurium. Res J Microbiol 7:344-352. doi:10.3923/jm.2012.344.352

Hu H, Lan R, Reeves PR (2002) Fluorescent amplified fragment length polymorphism analysis of *Salmonella enterica* serovar Typhimurium reveals phage-type-specific markers and potential for microarray typing. J Clin Microbiol 40:3406-3415. doi:10.1128/JCM.40.9.3406-3415.2002

Hu H, Lan R, Reeves PR (2006) Adaptation of multilocus sequencing for studying variation within a major clone: evolutionary relationships of *Salmonella enterica* serovar Typhimurium. Genetics 172:743-750. doi:10.1534/genetics.105.046466

Lan R, Stevenson G, Donohoe K, Ward L, Reeves PR (2007) Molecular markers with potential to replace phage typing for *Salmonella enterica* serovar *typhimurium*. J Microbiol Methods 68:145-156. doi:10.1016/j.mimet.2006.07.004

Lindstedt BA, Vardund T, Aas L, Kapperud G (2004) Multiple-locus variable-number tandem-repeats analysis of *Salmonella enterica* subsp. *enterica* serovar Typhimurium using PCR multiplexing and multicolor capillary electrophoresis. J Microbiol Methods 59:163-172. doi:10.1016/j.mimet.2004.06.014

Mather AE, Reid SWJ, Maskell DJ, Parkhill J, Fookes MC, Harris SR, Brown DJ, Coia JE, Mulvey MR, Gilmour MW, Petrovska L, de Pinna E, Kuroda M, Akiba M, Izumiya H, Connor TR, Suchard MA, Lemey P, Mellor DJ, Haydon DT, Thomson NR (2013) Distinguishable epidemics of multidrug-resistant *Salmonella* Typhimurium DT104 in different hosts. Science 341:1514-1517. doi:10.1126/science.1240578

McClelland M, Sanderson KE, Spieth J, Clifton SW, Latreille P, Courtney L, Porwollik S, Ali J, Dante M, Du F, Hou S, Layman D, Leonard S, Nguyen C, Scott K, Holmes A, Grewal N, Mulvaney E, Ryan E, Sun H, Florea L, Miller W, Stoneking T, Nhan M, Waterston R, Wilson RK (2001) Complete genome sequence of *Salmonella enterica* serovar Typhimurium LT2. Nature 413:852-856. doi:10.1038/35101614

Mikasová E, Drahovská H, Szemes T, Kuchta T, Karpíšková R, Sásik M, Turna J (2005) Characterization of *Salmonella enterica* serovar Typhimurium strains of veterinary origin by molecular typing methods. Vet Microbiol 109:113-120. doi:10.1016/j.vetmic.2005.05.006

Mmolawa PT, Schmieger H, Tucker CP, Heuzenroeder MW (2003) Genomic structure of the *Salmonella enterica* serovar Typhimurium DT 64 bacteriophage ST64T: evidence for modular genetic architecture. J Bacteriol 185:3473-3475. doi:10.1128/JB.185.11.3473-3475.2003

Muñoz N, Diaz-Osorio M, Moreno J, Sánchez-Jiménez M, Cardona-Castro N (2010) Development and evaluation of a multiplex real-time polymerase chain reaction procedure to clinically type prevalent *Salmonella enterica* serovars. J Mol Diagn 12:220-225. doi:10.2353/jmoldx.2010.090036

Ng LK, Mulvey MR, Martin I, Peters GA, Johnson W (1999) Genetic characterization of antimicrobial resistance in Canadian isolates of *Salmonella* serovar Typhimurium DT104. Antimicrob Agents Chemother 43:3018-3021

Pang S, Octavia S, Reeves PR, Wang Q, Gilbert GL, Sintchenko V, Lan R (2012) Genetic relationships of phage types and single nucleotide polymorphism typing of *Salmonella enterica* serovar Typhimurium. J Clin Microbiol 50:727-734. doi:10.1128/JCM.01284-11

Pedulla ML, Ford ME, Karthikeyan T, Houtz JM, Hendrix RW, Hatfull GF, Poteete AR, Gilcrease EB, Winn-Stapley DA, Casjens SR (2003) Corrected sequence of the bacteriophage P22 genome. J Bacteriol 185:1475-1477. doi:10.1128/JB.185.4.1475-1477.2003

Ross IL, Heuzenroeder MW (2005) Discrimination within phenotypically closely related definitive types of *Salmonella enterica* serovar Typhimurium by the multiple amplification of phage locus typing technique. J Clin Microbiol 43:1604-1611. doi:10.1128/JCM.43.4.1604-1611.2005

Rychlík I, Hradecka H, Malcova M (2008) *Salmonella enterica* serovar Typhimurium typing by prophage-specific PCR. Microbiology 154:1384-1389. doi:10.1099/mic.0.2007/015156-0

Stanley TL, Ellermeier CD, Slauch JM (2000) Tissue-specific gene expression identifies a gene in the lysogenic phage Gifsy-1 that affects *Salmonella enterica* serovar Typhimurium survival in Peyer's patches. J Bacteriol 182:4406-4413

Tanaka K, Nishimori K, Makino S-I, Nishimori T, Kanno T, Ishihara R, Sameshma T, Akiba M, Nakazawa M, Yokomizo Y, Uchida I (2004) Molecular characterization of a prophage of *Salmonella enterica* serotype Typhimurium DT104. J Clin Microbiol 42:1807-1812. doi:10.1128/JCM.42.4.1807-1812.2004
